# Treatment of Infections in Young Infants in Low- and Middle-Income Countries: A Systematic Review and Meta-analysis of Frontline Health Worker Diagnosis and Antibiotic Access

**DOI:** 10.1371/journal.pmed.1001741

**Published:** 2014-10-14

**Authors:** Anne CC Lee, Aruna Chandran, Hadley K. Herbert, Naoko Kozuki, Perry Markell, Rashed Shah, Harry Campbell, Igor Rudan, Abdullah H. Baqui

**Affiliations:** 1 Department of Pediatric Newborn Medicine, Brigham and Women's Hospital, Boston, Massachusetts, United States of America; 2 Department of International Health, Johns Hopkins Bloomberg School of Public Health, Baltimore, Maryland, United States of America; 3 Department of Health and Nutrition. Save the Children, Washington (D.C.), United States of America; 4 Global Health Academy and Centre for Population Health Sciences, The University of Edinburgh Medical School, Edinburgh, Scotland, United Kingdom; Umeå Centre for Global Health Research, Umeå University, Sweden

## Abstract

**Background:**

Inadequate illness recognition and access to antibiotics contribute to high case fatality from infections in young infants (<2 months) in low- and middle-income countries (LMICs). We aimed to address three questions regarding access to treatment for young infant infections in LMICs: (1) Can frontline health workers accurately diagnose possible bacterial infection (pBI)?; (2) How available and affordable are antibiotics?; (3) How often are antibiotics procured without a prescription?

**Methods and Findings:**

We searched PubMed, Embase, WHO/Health Action International (HAI), databases, service provision assessments (SPAs), Demographic and Health Surveys, Multiple Indicator Cluster Surveys, and grey literature with no date restriction until May 2014. Data were identified from 37 published studies, 46 HAI national surveys, and eight SPAs. For study question 1, meta-analysis showed that clinical sign-based algorithms predicted bacterial infection in young infants with high sensitivity (87%, 95% CI 82%–91%) and lower specificity (62%, 95% CI 48%–75%) (six studies, *n* = 14,254). Frontline health workers diagnosed pBI in young infants with an average sensitivity of 82% (95% CI 76%–88%) and specificity of 69% (95% CI 54%–83%) (eight studies, *n* = 11,857) compared to physicians. For question 2, first-line injectable agents (ampicillin, gentamicin, and penicillin) had low variable availability in first-level health facilities in Africa and South Asia. Oral amoxicillin and cotrimoxazole were widely available at low cost in most regions. For question 3, no studies on young infants were identified, however 25% of pediatric antibiotic purchases in LMICs were obtained without a prescription (11 studies, 95% CI 18%–34%), with lower rates among infants <1 year. Study limitations included potential selection bias and lack of neonatal-specific data.

**Conclusions:**

Trained frontline health workers may screen for pBI in young infants with relatively high sensitivity and lower specificity. Availability of first-line injectable antibiotics appears low in many health facilities in Africa and Asia. Improved data and advocacy are needed to increase the availability and appropriate utilization of antibiotics for young infant infections in LMICs.

**Review Registration:**

PROSPERO International prospective register of systematic reviews (CRD42013004586).

*Please see later in the article for the Editors' Summary*

## Abstract 2

Anne C. C. Lee and colleagues assess the factors affecting access to treatment for neonatal and infant infections in low- and middle-income countries by conducting a systematic review and meta-analysis of frontline health worker diagnosis and access to antibiotics.

*Please see later in the article for the Editors' Summary*

## Editors' Summary

**Background:**

Neonatal mortality—death that occurs during the first 28 days of life—accounts for nearly half of all the deaths that occur in children before they reach their fifth birthday. Worldwide, nearly 3 million neonatal deaths occur every year. Three bacterial infections—sepsis (infection of the bloodstream), pneumonia (infection of the lungs), and meningitis (infection of the brain's protective covering)—are responsible for nearly a quarter of all neonatal deaths. Babies born in low- and middle-income countries (LMICs) are at particularly high risk of developing neonatal bacterial infections because the risk factors for these infections, which include maternal infections and unhygienic delivery care, are more common in LMICs than in high-income countries. Babies born in LMICs are also at a high risk of dying from bacterial infections because access to appropriate medical care and antibiotics is often poor.

**Why Was This Study Done?:**

To reduce neonatal deaths from bacterial infections in LMICs, health care experts need to identify the factors that limit access to medical care and antibiotics in these countries. Are babies dying because health care providers fail to diagnose neonatal bacterial infections, because antibiotics are not available in first-line health facilities, or for some other reason? In this systematic review and meta-analysis, the researchers investigate access to treatment for neonatal bacterial infections in LMICs by first asking whether frontline health workers in LMICs can accurately diagnose bacterial infections in neonates and young infants (babies less than 2 months old). Next, they ask whether antibiotics for treating neonatal infections are available and affordable in LMICs. Finally, they ask how often antibiotics are procured for young children (children up to the age of 5 years) without a prescription. A systematic review uses pre-defined criteria to identify all the research on a given topic; meta-analysis uses statistical methods to combine the results of several studies.

**What Did the Researchers Do and Find?:**

The researchers identified 37 published studies, 46 surveys of drug availability and affordability in LMICs (Health Access International databases), and eight surveys of the capacity of health facilities in LMICs to provide quality health care services (service provision assessments) that met their inclusion criteria. Meta-analysis of six studies indicated that a combination of simple clinical signs for the diagnosis of bacterial infection in children predicted very severe disease in young infants with a sensitivity of 87% and a specificity of 62% (“sensitivity” indicates the percentage of true positives detected by a test; “specificity” indicates the percentage of healthy people that a test correctly identifies as healthy) compared to a physician's diagnosis with laboratory testing. Meta-analysis of eight studies indicated that frontline health workers (for example, community health workers) diagnosed very severe disease (including possible bacterial infection) in young infants with a sensitivity of 82% and a specificity of 69% compared to trained physicians. The national surveys analyzed indicated that first-level (primary) health facilities in Africa and South Asia had low, variable stocks of recommended first-line injectable antibiotics and that the cost of these drugs was high. By contrast, some oral antibiotics were widely available at low cost in most regions. Finally, meta-analysis of 11 studies indicated that, in LMICs, 25% of antibiotic purchases for the treatment of young children were obtained without a prescription.

**What Do These Findings Mean?:**

These findings suggest that trained frontline health workers should be able to identify most young infants who have possible bacterial infections in LMICs but may also diagnose bacterial infections in many young infants who are not infected. This may lead to the inappropriate use of antibiotics and facilitate the emergence of antibiotic resistance. These findings also show that the availability and affordability of first-line injectable antibiotics is low in many health facilities in Africa and Asia. The lack of neonatal-specific data on illness recognition, antibiotic formulations and availability, and other aspects of this systematic review and meta-analysis are likely to limit the accuracy of these findings. Nevertheless, the researchers suggest that, to decrease the neonatal death toll in LMICs, governments, policymakers, and the pharmaceutical industry need to work together to improve the diagnosis of neonatal bacterial infections and to increase the availability, affordability, and appropriate use of antibiotics for the treatment of these infections.

**Additional Information:**

Please access these websites via the online version of this summary at http://dx.doi.org/10.1371/journal.pmed.1001741.

- WHO provides information on global efforts to reduce global child mortality  and on ending preventable neonatal deaths  (available in several languages)
- The United Nations Children's Fund  (UNICEF) works for children's rights, survival, development, and protection around the world; it provides information on global efforts to reduce child mortality , and its Childinfo website provides detailed statistics about neonatal survival and health; its “Committing to Child Survival: A Promise Renewed” webpage includes links to its 2013 progress report and to videos about ending preventable child deaths
- The WHO has published a report entitled  UN Commission on Life Saving Commodities for Women and Children
- The Healthy Newborn Network (NHH) is an online community of more than 80 partner organizations that addresses critical knowledge gaps in newborn health; its website includes information on neonatal infections in LMICs
- Kidshealth, a resource provided by the not-for-profit Nemours Foundation, has information for parents on neonatal infections (in English and Spanish)
- The MedlinePlus Encyclopedia has a page on neonatal sepsis (in English and Spanish)
- A personal story about fatal neonatal bacterial meningitis is available on the website of Meningitis UK, a not-for-profit organization; the site also includes a survivor story

## Introduction

In 2010, there were an estimated 6.8 million cases of possible severe bacterial infection (pSBI), including 2.5 million cases of meningitis, pneumonia, sepsis or tetanus, diagnosed in neonates in South Asia, sub-Saharan Africa, and Latin America [1]. The incidence of neonatal infection ranges from 5.5 cases/1,000 live births for blood culture-confirmed infections in first-level facilities, to as high as 170 cases/1,000 births for clinically diagnosed pSBI in community-based settings [2]. Neonatal sepsis, pneumonia, and meningitis are the most common severe infections in the first month of life, and resulted in ∼718,000 neonatal deaths globally in 2010 [3]. Infections account for approximately 23% of neonatal deaths, yet as high as 50% in low-income settings [3],[4]. Numerous factors place newborns in low- and middle-income countries (LMICs) at higher risk for developing and dying from infections. Risk factors for neonatal infections, such as maternal infections, unhygienic delivery care, preterm birth, and intrauterine growth restriction are more prevalent in LMICs [5]. Furthermore, case fatality from neonatal infections is as high as 40% in LMICs, in large part due to poor access to appropriate medical care and antibiotics [5]–[8].

Timely and adequate diagnosis and treatment of bacterial infections with antibiotics are critical to reduce global neonatal and child mortality [9],[10]. Neonatal infections are difficult to recognize, even in high-resource settings, because their symptoms are non-specific and clinical infection is corroborated by positive cultures in only 3%–10% of suspected cases [2],[11],[12]. Diagnosis is challenging in low-resource settings where little or no capacity for etiological diagnosis or laboratory testing exists, and providers must often rely on clinical symptoms and algorithms alone. The extension of the WHO Integrated Management of Childhood Illness (IMCI) strategy to include newborns, coupled with the development and validation of clinical algorithms for young infants (<2 months) [13],[14], have been critical steps to improving the detection of very severe disease (VSD), including pSBI, in these settings. Case management of pSBI in first-level facilities and communities is feasible and may reduce neonatal mortality by 34%–62% [15]–[17]. In a systematic review of studies reporting the etiology of community acquired neonatal sepsis from LMICs, the common major pathogens were *Staphylococcus aureus* (26% of blood culture isolates), *Klebsiella* (21%), and *Escherichia coli* (8%), while Group B strep was uncommon (2%) [18], although regional differences have been noted [19]. Currently, WHO recommends as first-line treatment for neonatal sepsis injectable gentamicin and procaine benzylpenicillin for ten days, and as second line, ceftriaxone treatment for ten days [20]. These antibiotics are now on the WHO Model List of Essential Medicines for Children and have been targeted by the United Nations Commission for Life-Saving Commodities for Women and Children as key commodities to reduce neonatal mortality [21].

In order for governments and health systems to prioritize this high-impact, evidence-based intervention, it is imperative to understand the current landscape of access to antibiotics for treating neonatal infections in developing countries. Antibiotic access deserves special consideration in neonates, infants, and children, given their specialized dosing, drug formulations, delivery routes, risk profile, physiology, and monitoring needs [21],[22]. Furthermore, optimal antibiotic choice may vary regionally, depending on local pathogens, resistance patterns, drug availability, and cost [23].

**Figure 1:**
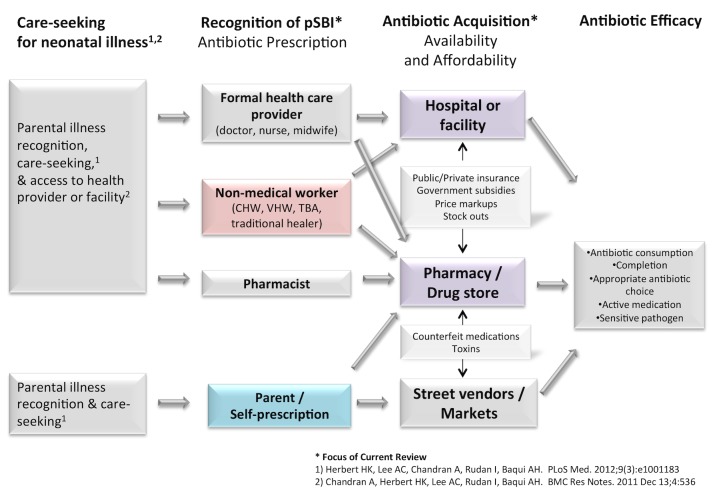
Conceptual model of access to antibiotics for newborns with infection.

The objective of this study was to review the broad landscape of factors affecting access to treatment for neonatal infections in LMICs, in order to identify key barriers and bottlenecks. We developed a conceptual framework to describe the potential pathways that may be taken from the point of illness recognition to the receipt and use of an antibiotic in a newborn (Figure 1). We have recently reviewed the literature on care-seeking patterns by caregivers for newborn illness [24], which is the first critical step required to access treatment for neonatal infections, as well as access to health facilities [25]. Once a caregiver suspects illness in a newborn and decides to bring the infant to a health provider, the provider must recognize whether an illness is a possible bacterial infection (pBI) requiring an antibiotic and subsequently prescribe the antibiotic. The prescriber may be a trained medical provider (e.g., a doctor, a nurse, or a midwife), a pharmacist, or a health worker who may or may not have been trained through the formal medical system (e.g., community health worker [CHW], traditional birth attendant, or traditional healer). Alternatively, parents may self-prescribe antibiotics where antibiotics are openly available over the counter. After being prescribed the antibiotic, factors influencing acquisition include antibiotic availability and affordability. Antibiotics may be obtained from pharmacies or within health facilities, either from the public or private sector, and informally from drug stores, street vendors, markets, or traditional healers. Government subsidies or health insurance may cover a substantial fraction or the entire cost of some medications in the public sector and help increase affordability particularly for the poor, while cost markup in the private sector may inflate prices and reduce affordability. After purchase, the parent must decide to give the antibiotic to the newborn. Finally, many factors may affect antibiotic utilization, such as antibiotic formulation, concentration, taste, and toxicities. 

For this analysis, we systematically reviewed the literature to answer the following specific study questions within the above described framework: (1) Can frontline health workers use clinical sign algorithms to accurately diagnose possible bacterial infection in young infants, as compared to physicians? (2) How available and affordable are antibiotics for treating neonatal infections in health facilities/pharmacies in LMICs? (3) What fraction of antibiotic purchases for treating neonatal/pediatric infections is accessed without prescription by a health provider (i.e., over-the-counter) in LMICs?

## Methods

### Literature Review and Data Sources

**Figure 2:**
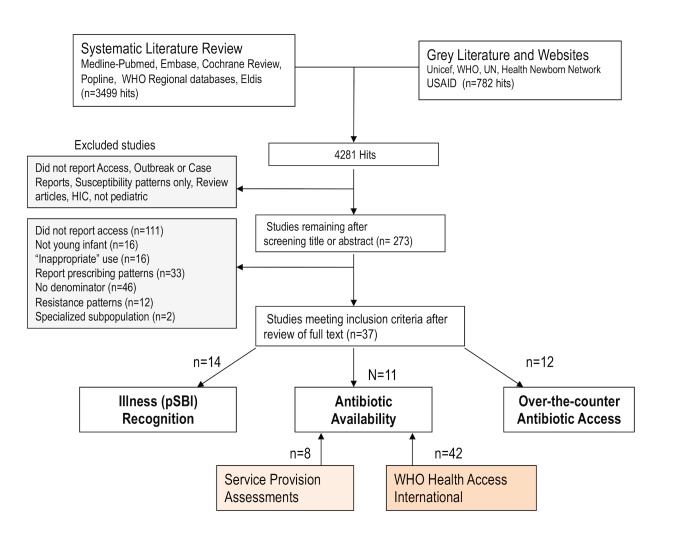
Search strategy and results for literature review of published and grey literature.

We conducted a systematic review of the published and grey literature, which was originally done between February and September 2010, updated in June 2013 and May 2014, with no date restrictions (Figure 2). The Preferred Reporting Items for Systematic Reviews and Meta-Analyses (PRISMA) statement for the systematic review is available in Text S1. The searches occurred in phases to address each of the study questions, and the detailed search strategy and terms are shown in Text S2.

Databases searched included PubMed, Embase, WHO regional databases (LILACS, IMEMRO, AIM), POPLINE, and Cochrane (review protocol available in Text S3). We also reviewed bibliographies of sentinel articles. Grey literature sources included Eldis, UN, UNICEF, and donor websites (USAID, Saving Newborn Lives, Healthy Newborn Network). Demographic and Health Surveys (DHS), Multiple Indicator Cluster Surveys (MICS), service provision assessments (SPAs), and WHO Health Action International (HAI) global databases were searched. SPAs are conducted in a nationally representative sample of >400 health facilities, including government, general, and specialized facilities as well as facilities in the non-governmental, private-for-profit, and non-profit sectors. Each SPA conducted includes a facility audit to assess infrastructure, equipment, and pharmacy. WHO HAI developed a standardized methodology for assessing drug availability and affordability in LMICs in 2003 [26]. In brief, data are collected from at least four different geographic areas in a nation, using a random cluster sample of public facilities and private retail pharmacies. In large countries, sub-national surveys are also conducted. Data collectors visit selected medicine outlets and obtained data on availability and pricing on a list of 30 WHO essential drugs on a particular day.

### Inclusion Criteria

There were no language restrictions. If potential articles of interest were identified in non-English languages, the abstract was translated to English via Google Translate to determine whether the article met inclusion criteria. Articles were considered for inclusion if they were from LMICs, as defined by the World Bank, and reported data to inform one of the research questions. We included searches for specific infections (sepsis, meningitis, pneumonia, urinary tract infection, omphalitis) as well as broader terms for the clinical diagnosis of infection (IMCI, algorithms, possible serious bacterial infection). We included articles that reported on pBI of any severity, including pSBI (sepsis, meningitis, pneumonia) as well as local bacterial infection (omphalitis or skin infections). A neonate was defined as <28 days of age and a young infant <2 months of age. For study question 1, the study population included newborns and young infants. For study question 2, we included availability and affordability data on common first- and second-line injectable antibiotics and formulations for treating neonatal infections including ampicillin, penicillin or procaine benzylpenicillin, gentamicin, and ceftriaxone, and also extracted data on oral amoxicillin and cotrimoxazole [22],[27]. For oral agents, we extracted data on suspension formulation when available, or capsular formulation given the potential to re-suspend in fluid for neonates. For study question 3, we determined *a priori* to include data on the pediatric population less than five years of age, given the likely paucity of neonatal specific data.

### Exclusion Criteria

We excluded studies that reported on adult populations and those solely focusing on the inappropriate use of antibiotics. The goal of this review was to populate a model parameter for “the proportion of neonates with possible bacterial infection accessing antibiotics” for a modeling exercise that is described elsewhere (Rudan I, personal communication). Thus, studies that reported on antibiotic prescription patterns or utilization without a denominator to inform this parameter were excluded (i.e., studies reporting numbers of antibiotic prescriptions with no denominator, or per WHO/INRUD methodology [antibiotic prescriptions per patient encounter, population, or total prescriptions]) [28]. We excluded studies from specialized sub-populations or high-income countries, individual case reports, duplicate studies, and studies reporting on outbreaks or susceptibility patterns alone.

### Data Abstraction

Data were abstracted into a standard excel file developed for Child Health Epidemiology Reference Group (CHERG) reviews [29] by two independent reviewers. Data regarding population characteristics, population selection, study design, setting, age range, case definitions of possible infection, gold/reference standard diagnosis, antibiotic prescription, availability, or cost were recorded. For study question 1 (the accuracy of pBI diagnosis), a two-by-two table was constructed for each study to determine the true positives, false positives, true negatives, and false negatives, comparing clinical algorithm to the gold standard reference definition. In validation studies, if the performance of more than one algorithm was presented, the “best” algorithm chosen by the authors (typically the one with the highest sensitivity) was used in the analysis. For study question 2, the proportion of medicine outlets with specified antibiotics for treating neonatal infection were recorded, and the number of outlets (pharmacies or facilities surveyed if available). From the WHO HAI database, the following data were abstracted regarding neonatal antibiotic formulations: availability (percent of venues that carry a medication on the day of data collection), pricing (unit price in US dollars), and affordability (number of days of work required by the lowest paid unskilled government worker to purchase a course of antibiotic treatment). For study question 3, data were abstracted on the overall number of pediatric antibiotic purchases or courses over a specified time period, and the number (or proportion) of those that were obtained by self-medication, or without a health provider prescription.

### Study Quality Assessment

For study question 1 (accuracy of pBI diagnosis), methodological quality was assessed per the Cochrane Diagnostic Test Accuracy Working group recommendations [30] using the QUADAS-2 (Quality Assessment of Diagnostic-Accuracy Studies-2) [31]. For study questions 2 and 3, the individual and overall study quality was assessed based on a modification of GRADE [32] methods for systematic reviews described by CHERG, using principles relevant for the aims of this particular review [29]. Individual studies were evaluated for limitations and biases in the following domains: study design, population selection and representativeness, definitions, precision, and generalizability to the population of interest [24]. For each of these domains, a score was assigned (0, no limitations; 1, some limitations; and 2, serious limitations). A total quality assessment score was given to each study. Study design was scored according to whether data were (a) prospectively or retrospectively collected, and potentially influenced by recall bias, (b) directly observed versus self-reported (reporting bias), and by (c) potentially biased by high rates of losses to follow-up. Population representativeness described the extent to which the study sample was representative of the general population as being either population or health facility-based with minimal or moderate bias. Quality of definitions described the extent to which study defined and characterized the parameter of interest. Consistency was assessed across all studies to ascertain the extent to which these definitions were similar. Generalizability was defined according to the degree to which results could be applied to our target population of interest (newborns in LMICs). Precision was defined as the extent to which the study populations included a sufficient sample size. If a study's total study population was less than 50, the quality of the evidence was considered insufficient to generalize the effect of the outcome to the target population [33]. All studies were graded independently by two reviewers, and discrepancies in scoring were discussed and resolved. The study database and quality assessment are in Table S1.

### Data Analysis

#### Study question 1: diagnosis of pBI in young infants

Coupled forest plots were generated with Review Manager 5.1. Pooling of sensitivity and specificity separately fails to account for the inter-relatedness of the measures. Hierarchal bivariate models are recommended for meta-analysis [30] and were analyzed using the Stata 12.0 “metandi” command, and hierarchal summary receiver operating characteristic curves were generated with the “metanplot” command. Sub-group analysis was conducted by health worker type and gold standard reference diagnosis type (clinical versus laboratory/radiologic evidence). Subgroups with fewer than four studies were pooled by univariate random effects (“metan” command) given the failure of the metandi command to converge with less than four studies. Meta-regression was conducted using the Stata “metareg” command, and logit transformed proportions and standard errors were calculated to explore the significance of sources of heterogeneity (type of health worker, reference standard diagnosis, location [community versus facility]).

#### Study question 2: antibiotic availability, pricing, and affordability

Given the differing methodology and sampling frame from the studies identified in the literature, WHO HAI Project, and SPAs, the survey data from different sources were not combined and are reported separately. From the HAI database, national survey data on availability, pricing, and affordability were grouped and analyzed by WHO major world regions. If a particular country had data from more than one year, we used the most recent survey data; if multiple sub-national surveys were conducted, we calculated a national mean for the country. For availability, we used data from the brand (originator, most sold generic or lowest price generic) of highest availability for the survey. Antibiotic pricing was calculated for treating a 3 kg newborn for a ten-day treatment course based on recommended neonatal dosing and duration from several recent reviews [22],[27],[34]. Affordability data were available from the WHO HAI database for cotrimoxazole and amoxicillin suspensions. For injectable antibiotics, affordability was calculated by dividing the price of the full course of antibiotic by the daily wage of the lowest paid government worker. For each WHO region sub-group, the median data point and range were calculated for each indicator (availability, price, and affordability).

#### Study question 3: non-prescription antibiotic use

Proportions and standard errors were logit transformed, and meta-analysis was conducted with random effects, assuming that the true prevalence of non-prescription antibiotic use may vary between studies. The Higgins I^2^ statistic and 95% confidence intervals were calculated. Meta-regression was conducted to explore sources of heterogeneity (age group, region). Stata 12.0 was used for these analyses.

### Registration

The review was registered in the PROSPERO International prospective register of systematic reviews (CRD42013004586).

## Results

In the literature review, a total of 2,554 titles were identified, and after reviewing titles and/or abstracts, we retrieved 261 full articles (Figure 2), of which 37 met study criteria. These results and findings from SPAs and HAI databases are reported within each study question.

### Study Question 1. Diagnosis of pBI in Young Infants

We identified a total of 14 studies in the literature that reported on health worker diagnosis of pBI (Table 1). Eleven studies reported on a population of young infants (<2 months) with four of those studies excluding <7 day old infants, and three which reported on neonates (<1 month). In SPA and DHS surveys, there were no data identified on pBI diagnosis in young infants. None of the studies indicated the proportion of preterm or low birth weight babies in the validation sample. Nine assessments were conducted in facilities (outpatient clinics, primary care clinics, or emergency department assessments), and five were conducted within the community. Six studies reported on the accuracy of clinical-sign-based diagnosis of pBI by varying IMCI algorithms compared to a gold standard diagnosis by an expert physician, which included laboratory and radiologic testing. The remaining compared IMCI classification between first-level, frontline health workers and physicians. Health workers ranged from CHWs (*n* = 4), first-level facility-based health workers (*n* = 6), to pediatricians (*n* = 3). Five studies reported on the diagnosis of severe illness (pSBI or VSD) and the remaining reported on any pBI (“need for referral,” “yellow OR red zone” on IMCI).

**Table 1:** Literature review: studies of diagnosis of possible bacterial infection/severe disease in newborns and young infants (<59 days).

Author (Publication Year)	Setting	Health Worker	Patient Population	Clinical Algorithm for Illness	Gold Standard Definition	*N* Young Infants Assessed	*N* (Gold Standard) Infection/Severe Disease	Type Of Infection Or Severe Disease (if Indicated)^a^	Sensitivity (%)	Specificity (%)
**Kalter [59] (1997)**	Outpatient department and Emergency room of Children's HospitalBANGLADESH	Study pediatrician	7 days–2 months	IMCI guidelines 1995 - requiring referral (possible serious bacterial infection, diarrhea with severe dehydration, not able to feed)	Pediatrician-assessed severe illness requiring hospital admission, confirmatory laboratory testing available	234	105	101 pneumonia, 19 local bacterial infection, 16 septicemia, 16 watery diarrhea or dysentery, 4 meningitis	84	54
**Gupta [60] (2000)**	Outpatient department and Emergency room of Medical College hospital INDIA	Pediatric trainee	1 week–2 months	IMCI algorithm 1997 (requiring referral)	Diagnosis of serious bacterial infection by supervising physician/attending diagnosis with relevant laboratory investigation	105	57	31 diarrhea, 21 septicemia, 16 pneumonia, 10 meningitis	97	60
**WHO Young Infant Study [38] (2003)**	Outpatient clinics and first level hospitals ETHIOPIA, GAMBIA, PAPUA NEW GUINEA, PHILIPPINES	Pediatrician or study nurse	<2 months	Presence of 1 out of 14 signs	Severe disease (sepsis, meningitis, hypoxemia, or radiologic pneumonia)	3,303	718	346 pneumonia or mild hypoxemia, 372 bacteremia , 34 meningitis, 259 severe hypoxemia	87	54
**English [61] (2004)**	Outpatient ill consultations, Kilifi District Government Hospital KENYA	Clinical staff using IMCI guidelines	(a) <7 days(b) 7–59 days	IMCI algorithm 1999 (16 signs)	Admitting physician diagnosis with confirmatory laboratory testing	(a) 329(b) 897	(a) 120(b) 124	Outpatient (38% acute respiratory infection, 14% skin infection, 8% conjunctivitis). Inpatient (32% severe infection/pneumonia, 15% neonatal sepsis)^b^	(a) 94(b) 97	(a) 25(b) 45
**Goswami [62] (2006)**	Outpatient department and Emergency room of Medical College tertiary care hospital INDIA	Facility based first-line health worker	(a) 0–7 days(b) 7–59 days	IMCI algorithm 2002	Pediatrician evaluation requiring hospital admission, including laboratory investigation as required	249	(a) 195(b) 107	NS	(a) 79(b) 85	(a) 79(b) 78
**Young Infant Clinical Signs [39] (2008)**	Outpatient clinics (primarily medical school/teaching hospitals, 3 primary health clinics)BANGLADESH	Facility based primary health worker	(a) <7days(b) 7–59 days	7-sign IMCI algorithm	Severe illness requiring urgent hospitalization diagnosed by expert pediatrician, confirmatory lab testing	8,889	1,132	(a) 258 severe infection (sepsis, meningitis, pneumonia), 105 preterm-LBW, 143 asphyxia(b) 400 severe infection (sepsis, meningitis, pneumonia), 18 preterm-LBW, 3 asphyxia	(a) 88(b) 79	(a) 75(b) 79
**Baqui [41],[63] (2009)**	Rural Sylhet, community-basedBANGLADESH	CHWs	<28 days	PVSD (20 sign) or VSD (8 sign) algorithm adapted from Bangladesh IMCI	Newborns with VSD or possible very severe (PSVD) disease by N-IMCI identified by physicians	288	VSD 74VSD or PVSD 133	NS	(a) VSD 91(b) VSD/PVSD 87	(a) VSD 95(b) VSD/PVSD 87
**Bhattacharyya [64] (2009)**	Medical College, KolkataINDIA	Facility based first-line health worker	<2 months	IMNCI algorithm 2005 - red zone or yellow zone (12 signs for urgent referral)	Pediatrician decision to investigate, admit/treat	117	(a) Red zone 45 (b) Red or yellow 59	NS	(a) 88(b) 73	(a) 72/(b) 41
**Darmstadt [65] (2009)**	Rural Mirzipur, served by private Kundimini hospitalBANGLADESH	CHWs	<28 days	VSD (11 sign) or PVSD (17 sign algorithm adaptation of Bangladesh IMCI)	Newborns with very severe or possible VSD by N-IMCI identified by physicians	395	(a) VSD 11(b) VSD or PVSD 38	NS	(a) VSD 73(b) VSD/PVSD 45	(a) VSD 98(b) VSD/PVSD 95
**Kaur [66](2010)**	Outpatient department/ED of medical college INDIA	Facility based first-line health worker	<2 months	IMNCI algorithm requiring referral 2005	Serious bacterial infection diagnosed by senior pediatrician	419	213	NS	89	57
**Shewade [67] (2012)**	Outpatient Community Health Center/RaipuraniINDIA	IMNCI trained health worker (ANM/AWW)	<2 months	IMNCI algorithm 2006 (red or yellow zone), requiring referral	Investigator/Pediatrician diagnosis by IMCI criteria (yellow-red zone)	26	(a) Red zone 10(b) Red or yellow 15	NS	(a) 30(b) 47	(a) 100(b) 100
**EXCLUDED from Meta-analyses**										
**Bang [37] (2005)**	Rural Gadichiroli, community-basedINDIA	CHWs	<28 days	Weak cry, suck, unconscious, baby cold to touch or fever, skin/umbilical infection, abdomen distension/vomiting, chest indrawing (2 or more criteria)	Newborns with clinical criteria for infections by computer algorithm	5,268	552	NS	NS	NS
**Biswas [35] (2011)**	Random selection of frontline IMNCI provider assessments, community-based West BengalINDIA	Frontline community worker (Auxiliary nurse midwife, AWW)	<2 months	IMNCI algorithm	IMNCI classification by gold standard faculty physicians		52	NS	33.6	NS
**Khanal [36] (2011)**	Rural Morang district, community-based eastern Nepal NEPAL	Female community health volunteers	<2 months	MINI algorithm (any 1 unable to feed, lethargic, respiratory rate >60, severe chest indrawing, grunting, T>37.5|<35.5, umbilical redness, >10 skin pustules, weak cry)	CHW assessment of signs of pSBI		1,051	NS	NS	NS

a Some studies did not report the exact breakdown of the bacterial infection diagnoses, but reported more generally on the illnesses detected. Hence, the *n*'s here do not necessarily add up to the *n* of those diagnosed as a bacterial infection by the gold standard.

b **Data extracted from English 2003, which evaluated the same population but did not have the same exact sample size [68].

ANM, auxiliary nurse midwife; AWW, Anganwadi worker; NS, not stated.

The individual study quality assessment is in Table S1, and the overall QUADAS-2 summary assessment is shown in Figure S1. There was high risk of bias in patient selection in eight studies, and unclear risk in three out of 14 studies. The blinding of the reference classification to the index assessment for pBI was unclear in nine studies; however, in all studies the index assessment was blinded to the gold standard. Eleven studies were included in the meta-analyses (including 15,499 neonates, 3,016 possible infection cases) (Table 2). Three studies were excluded from meta-analyses because they did not have sufficient data from which to calculate sensitivity/specificity [35],[36] or the gold standard was a computer algorithm and not physician diagnosis [37].

**Table 2:** Diagnostic accuracy of clinical algorithms and frontline health workers to detect severe disease/possible bacterial infection in young infants.

Outcome	Number of Studies	Number of Screened Infants	Cases of Possible Infection Detected by Health Workers	Sensitivity (95% CI)	Specificity (95% CI)
**Reference Gold Standard**					
Physician diagnosis with laboratory or radiologic testing/results	6	14,254	2,558	86.8% (81.8–90.6)	62.3% (48.0–74.9)
Physician clinical diagnosis only	5	1,245	458	76.6% (55.6–89.6)	83.5% (56.8–95.2)
**Frontline Health Workers (** ***n*** ** = 8 studies)**					
All frontline health workers (CHW and first level facility health worker)	(a) VSD 5(b) PVSD/VSD 8	11,857	(a) VSD 1,272(b) PVSD/VSD 2,136	(a) VSD: 80.1% (70.9–89.2)(b) PSVD/VSD 82.0% (75.7–88.2)	(a) VSD: 86.3% (72.6–100)(b) PSVD/VSD 68.5% (54.5–82.5)
First-level facility-based worker	(a) VSD 3(b) PVSD/VSD 6	11,174	(a) VSD 1,187(b) PVSD/VSD 1,965	(a) VSD: 72.5% (55.7–89.3)(b) PSVD/VSD 85.2% (78.7–91.7)	(a) VSD: 77.5% (75.8–79.1)(b) PSVD/VSD 59.2% (39.3–79.1)
CHW	2	683	(a) VSD 85(b) PVSD/VSD 171	(a) VSD: 86.8% (71.6–100)(b) PVSD OR VSD: 66.4% (25.8–100)	(a) VSD: 97.1% (94.1–100)(b) PSVD OR VSD: 91.3% (83.9–98.7)

For VSD (very severe disease), we are including red zone IMCI and pSBI (possible severe bacterial infection).

For PVSD (possible very severe disease), we are including both red AND yellow zone in IMCI, and pSBI as well as possible local bacterial infection.

#### Can clinical sign algorithms accurately predict severe disease in young infants?

The WHO Young Infants Study (YIS) was the sentinel multi-country study (1990–1993) from four countries (Ethiopia, Gambia, Papua New Guinea, Philippines) that informed the development of IMCI guidelines for the management of sick young infants in first-level facilities [38]. In the analysis of young infants <60 days (*n* = 3,303), the presence of one of 14 clinical signs assessed by a pediatrician or a study nurse predicted severe bacterial infection (sepsis, meningitis, hypoxemia, or radiologic proven pneumonia) in infants <2 months with a sensitivity of 87% and specificity of 54% [38]. While some clinical signs were more predictive of specific types of infections (i.e., bulging fontanelle or convulsions for meningitis, or chest indrawing for pneumonia), a single algorithm was chosen to indicate any pSBI given the overlapping non-specific signs in young infants and low prevalence of individual conditions. Serious bacterial infections were detected in 11% of these infants (meningitis [*n* = 35], sepsis [*n* = 120], and pneumonia [*n* = 259]) and the common isolated pathogens were *S. aureus*, *Streptococcus pneumonia*, and *E. coli*, with *E. coli* gram negatives more important in the first week of life. In Kilifi District Hospital, Kenya, English and colleagues reported that the 16-sign IMCI guidelines for infants 7–59 days predicted very severe illness with a sensitivity of 97% and specificity of 44%, and for infants <7 days with sensitivity 94% and specificity 25%. The Young Infant Clinical Signs Study enrolled 8,889 children from five countries (Bangladesh, Bolivia, Ghana, Pakistan, South Africa) [39]. This study aimed to identify any severe illness in young infants requiring hospital admission, and was not limited to severe bacterial infections alone. Among those babies who required admission among the study infants, the conditions varied between study sites with rates of severe infection (sepsis, meningitis, pneumonia) ranging from 23% in Bolivia to 70% in Pakistan among infants 7–59 days. Prematurity/low birth weight was the cause of admission for 0% (Bolivia) to 17% (Bangladesh) of infants in the first week, while birth asphyxia accounted for 0% (Bolivia) to 41% (Bangladesh). After this study, the WHO IMCI guidelines adopted a simpler algorithm including seven signs. When performed by primary health workers, the seven-sign algorithm predicted severe illness requiring hospital-level care with a sensitivity/specificity of 85%/75% (0–7 days old) and 74%/79% (7–59 days old) [39]. Of interest, the sensitivity of the algorithms was lower in the two African countries in this study (Ghana, South Africa).

**Figure 3:**
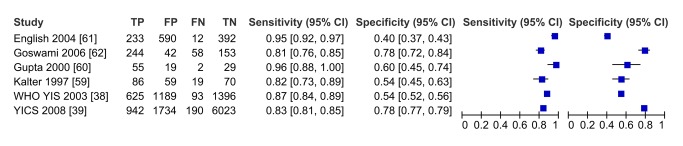
Forest plot of studies of diagnostic accuracy of clinical sign (IMCI) algorithms to detect severe disease/pBI in young infants compared to physician-laboratory diagnosis.

**Figure 4:**
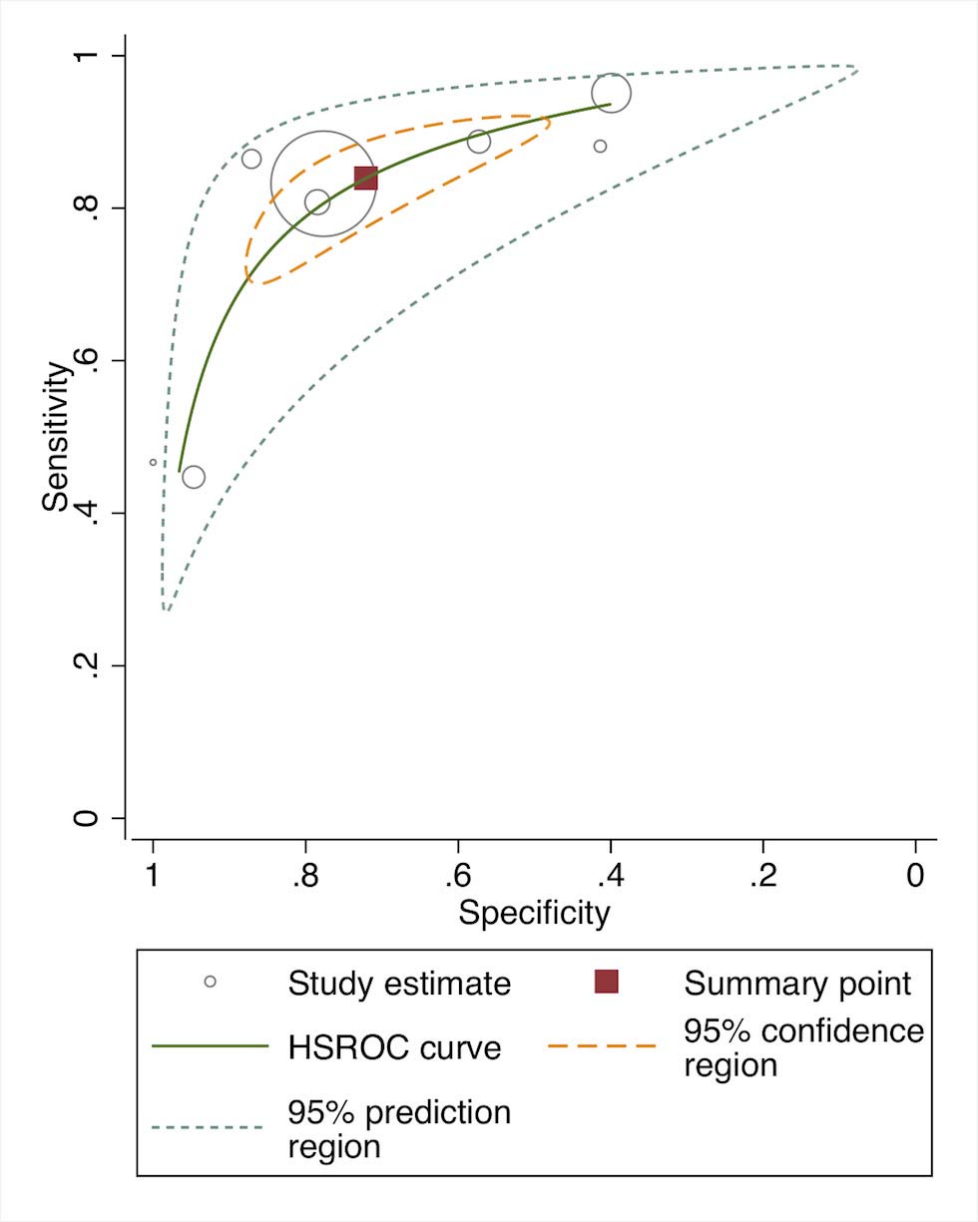
Receiver operating curve of studies of diagnostic accuracy of clinical sign algorithms versus physician-laboratory diagnosis of severe disease/pBI in young infants.

In meta-analysis, including six studies that validated diagnosis of severe disease by IMCI algorithms compared to gold standard reference of physician diagnosis including corroborating laboratory data (blood count, culture, chest X-ray, and/or cerebral spinal fluid), the average sensitivity was high (86.8%; 95% CI 81.8–90.6) and specificity was lower (62.3%; 95% CI 48.0–74.9) (Figure 3, forest plot; Figure 4 receiver operating curve; Table 2). Meta-regression showed significantly lower specificity of IMCI algorithms, when the gold standard reference definition included laboratory/radiologic data or was performed in the facility (with more frequent laboratory/radiologic capacity).

While the diagnostic accuracy of algorithms may vary in premature babies, who often have similar signs/morbidity as babies with infection (i.e., jaundice, poor feeding), none of the studies provided data on the proportion of babies screened who were either preterm or low birth weight, or stratified the validation study by these subgroups.

#### Can frontline health workers use clinical algorithms to accurately identify severe disease/possible bacterial infection?

The eight studies reporting on the validity of frontline health workers to diagnose pBI compared to physician diagnosis (with or without laboratory confirmation) are shown in Table 1. The six facility-based studies (Table 1) were IMCI evaluations (four India, one Kenya, one multi-country) with sensitivity/specificity ranging from 47%/100% for the classification of “red or yellow” zone disease requiring referral in a small evaluation in Raipurani, India to 96%/40% for a large assessment of the 16-sign IMCI guideline in Kilifi, Kenya. Among the community-based studies, two intervention studies from Bangladesh validated CHW classification of newborns by modified Bangladeshi IMCI criteria compared to physician classification, reporting that 73%–91% of cases of VSD were recognized by CHWs, with specificity ranging 95%–98% [40],[41].

**Figure 5:**
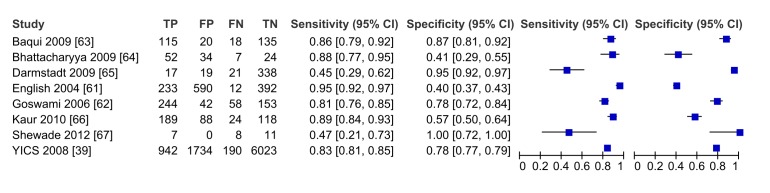
Forest plot of studies of diagnostic accuracy of frontline health worker diagnosis of pBI compared to physician diagnosis.

**Figure 6:**
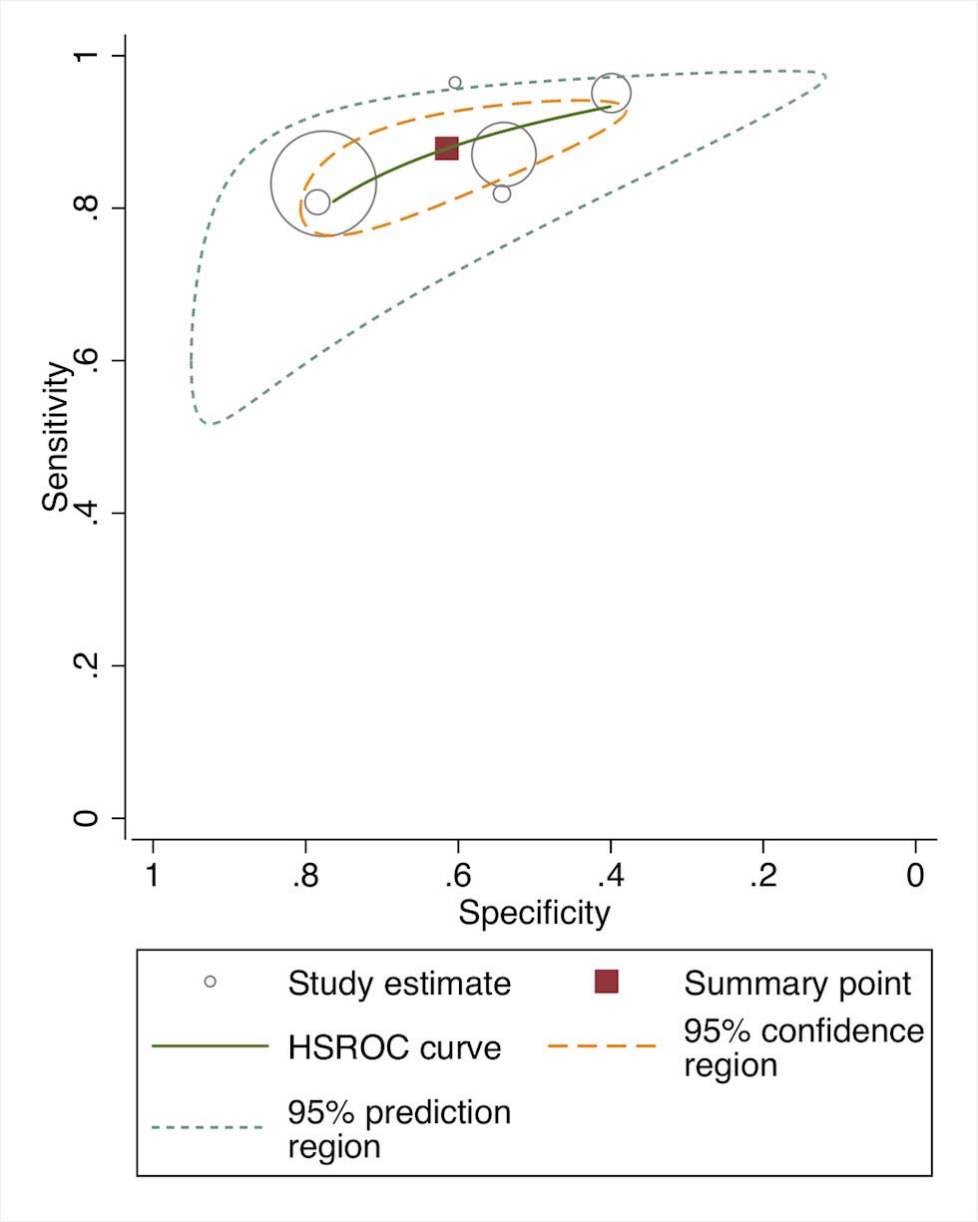
Receiver operating curve of studies of diagnostic accuracy of frontline health worker diagnosis of pBI compared to physician diagnosis.

In the pooled analysis, among all frontline health workers (facility and community-based), the sensitivity (80.1% [95% CI 70.9–89.2]) and specificity (86.3% [95% CI 72.6–100]) for the recognition of severe illness (VSD/pSBI) was high (five studies) (Table 2). For the recognition of all pBI (VSD and PVSD), the sensitivity was similar, but specificity was lower at 68.5% (eight studies, 95% CI 54.5–82.5) (Figure 5, forest plot; Figure 6, receiver operating curve). Meta-regression showed no difference in sensitivity by health worker type; however, specificity was significantly modified by the effect of health worker type. For facility-based health workers, the lower specificity was likely due to the higher availability of laboratory testing to corroborate the reference diagnosis. Most studies were graded as having risk for bias in patient selection given that many studies occurred in health facilities where parents presented with their sick children. Only one study was graded as being population-based, with low risk of-bias in patient selection.

Although three community-based studies provided relevant data, they were not included in the meta-analysis because they did not report sensitivity and specificity. In the SEARCH trial in Gadichiroli, India, village health workers could identify individual signs of neonatal illness in high agreement with physicians (mean 92.7% agreement on 46 variables) [42], and diagnosed 89% of cases meeting clinical sepsis criteria compared to a computer diagnostic algorithm based on neonatal symptoms [37]. In Nepal, female community health volunteers had high levels of agreement on the major signs of neonatal sepsis compared to facility-based CHWs (kappa >0.71 on ten signs) [36]. In Purulia, India, a program evaluation of government Integrated Management of Neonatal and Childhood Illnesses (IMNCI) training assessed frontline health worker performance [35]. Faculty members who conducted observation of home visits found that while a majority of infants had individual signs/symptoms assessed, the full IMNCI assessment was completed in only 32% of cases. Among those cases, 34.8% had the correct classification in all subgroups and 34.2% in at least one subgroup.

### Study Question 2. Antibiotic Availability

We identified data on availability of antibiotics for treating neonatal infections from three main sources: published literature (11 studies), WHO/HAI database (46 national surveys), and SPAs (eight national surveys).

The surveys reported in the published literature were typically of smaller sample size (<50 outlets), at the regional level (provincial or district), and of varying sampling methodology. One study reported on availability of pediatric formulations (i.e., suspension/syrup) [43]. Overall the study quality of data reported in the literature was low and heterogeneous (Table S1), and the availability of individual antibiotics varied widely between surveys (Table 3). The data were therefore not pooled and overall ranges are reported.

**Table 3:** Published literature on availability of first and second-line antimicrobials for treating neonatal infections.

Author (Publication Year)	Country	WHO Region	Rural/Urban	Sample	Parenteral				Oral	
					Percent Ampicillin	Percent Penicillin^a^	Percent Gentamicin	Percent Ceftriaxone	Percent Amoxicillin	Percent Cotrimoxazole Tab (Syrup)^b^
**Carasso [43] (2009)**	Ethiopia	AFR	Rural	5 budget pharmacies	80%					100% (40%)
**Carasso [43] (2009)**	Ethiopia	AFR	Rural	4 special pharmacies	100%					75% (75%)
**Gabra (2000) [69]**	Uganda	AFR	Mixed	20 facilities from four districts, ranging from lowest to highest-level facilities			27%		18%	59% 120 mg/tab92% 480 mg/tab
**Simoes [70](2003)**	Uganda, Tanzania, Niger	AFR	Mixed	First level health facilities (39 health centers Uganda; 7 Mpwapwa, Tanzania; 16 Boboye/Birninkonni Niger)		94%				
**Bartoloni [71](1998)**	Bolivia	AMR	Rural	10 district pharmacies, Camiri	100%		100%		100%	50%
**Cheraghali [72] (2005)**	Iran	EMR	Mixed	20 public primary health centers, Fars, Tehran, Khorasan, Khuzestan, Kermanshah					50%	21%
**Hafner (2002) [73]**	Kazakhstan	EUR	Urban	21 pharmacies (11 from center of city, 10 from suburbs) (2 pubic, 19 private)	80%–100%				20%–40%	60%–80% 120 mg/tab80%–100% 480 mg/tab
**Kotwani [74](2007)**	India	SEAR	Mixed	Public and private facilities (433 total) Chennai, Haryana, Karnataka, Maharashtra, West Bengal					75%	32%
**Kotwani (2013) [56]**	India	SEAR	Urban	80 public facilities (68 primary, 10 secondary, 2 tertiary)	11%		5%	6%	45%	33%
**Zhang [75] (2007)**	China	WPR	Rural	74 health facilities	91%		98%	76%	78%	90%
**Yang [76] (2009)**	China	WPR	Rural	36 pharmacies (18 public, 18 private) Hubei province				90%	95%	5%
**Overall Range**					11%–100%		5%–100%	6%–90%	18%–100%	5%–100%

Percent indicates the percentage of facilities or pharmacies surveyed with the antibiotic in stock at the time of the survey.

a Formulation of Penicillin was not provided.

b Sulfamethoxazole and trimethoprim.

AFR, WHO African Region; AMR, Region of the Americas; EMR, Eastern Mediterranean Region; EUR, European Region; SEAR, Southeast Asia Region; WPR, Western Pacific Region.

From the WHO/HAI database, the regional summary is shown in Table 4 with country-level data in Table S2. Ampicillin data were limited, though the drug was highly available in four African surveys (1 g/vial injection). Procaine benzylpenicillin (4 MIU/vial) was available in >90% of Ethiopian medicine outlets (2004), however in Haiti (2010), the 1 MIU preparation had very low availability at <10%. Injectable gentamicin had low-moderate median availability in the public sector in most regions (47%–68% of outlets, except Western-Pacific), with higher availability in the private sector (72%–96%). Ceftriaxone was generally less available, particularly in Africa where a median of 25% of public facilities had it in stock. Oral amoxicillin (capsules/tablets) and cotrimoxazole suspension were highly available (>75%) in both the public and private sector in most regions. The limited data on amoxicillin suspension (250 mg/5 ml, *n* = 14 surveys) showed high availability (>90%) in Africa and Eastern Mediterranean regions (Table S2).

**Table 4:** WHO/HAI data on antibiotic availability by WHO region.

WHO Region	Injectable						Oral			
	Ampicillin^a^ (1 g/Vial)		Gentamicin^b^ (40 mg/ml)		Ceftriaxone^c^ (1 g/Vial)		Amoxicillin^d^ 250 mg Capsules/Tablets		Cotrimoxazole^e^ Suspension (40 mg Trimethoprim+200 mg Sulfamethoxazole per 5 ml)	
	Public	Private	Public	Private	Public	Private	Public	Private	Public	Private
AFR	94 (42–100)	86 (71–90)	68 (27–100)	66 (19–96)	24 (10–64)	50 (11–100)	75 (67–93)	89 (18–100)	70 (15–94)	84 (33–100)
AMR	32	63	38 (15–60)	72 (60–83)	76 (31–100)	77 (49–91)	85	46 (31–62)	69 (5–98)	85 (63–93)
EMR			67	81	29 (11–100)	84 (12–100)	84 (20–100)	84 (36–100)	87 (20–100)	96 (40–100)
SEAR			47	77 (58–97)	70 (32–95)	34 (33–72)	76 (60–100)	83 (83–91)	75 (41–92)	64 (43–88)
WPR			100	96	72 (46–85)	31 (3–57)	83 (34–100)	88 (73–100)	25 (32–35)	33 (3–51)

Availability defined as the percentage of medicine outlets surveyed with the particular antibiotic in stock at the time of the survey. Data are presented as median and range for data available from the respective WHO region. Data on procaine benzyl penicillin only available from 1 survey (Haiti).

a Data based on national surveys from: AFR (*n* = 4), AMR (*n* = 1).

b Data based on national surveys from: AFR (*n* = 4), AMR (*n* = 3), EMR (*n* = 1), SEAR (*n* = 2), WPR (*n* = 1).

c Data based on national surveys from: AFR (*n* = 15), AMR (*n* = 9), EMR (*n* = 13), SEAR (*n* = 3), WPR (*n* = 4).

d Data based on national surveys from: AFR (*n* = 8), AMR (*n* = 2), EMR (*n* = 9), SEAR (*n* = 4), WPR (*n* = 5).

e Data based on national surveys from: AFR (*n* = 15), AMR (*n* = 9), EMR (*n* = 13), SEAR (*n* = 4), WPR (*n* = 5).

AFR, WHO African Region; AMR, Region of the Americas; EMR, Eastern Mediterranean Region; EUR, European Region; SEAR, Southeast Asia Region; WPR, Western Pacific Region.

National SPA surveys provided data on antibiotic availability based on pharmacy audits conducted in eight countries (Table 5). Injectable ampicillin, gentamicin, and ceftriaxone had low median availability in both delivery and primary child care facilities in Africa and Southeast Asia—available in fewer than half of facilities. In Africa, injectable penicillin was highly available in ambulatory pediatric facilities (>80%) although there were no data on specific formulation of procaine benzyl penicillin. The availability of oral antibiotics was much higher (>70%), although the availability of pediatric suspensions was low. Similarly, in surveys in Bangladesh and Egypt, first-line injectable antibiotic availability for treating neonatal sepsis was low.

**Table 5:** Service provision assessments: antibiotic availability in delivery and pediatric ambulatory facilities.

Region/Country	Delivery Facilities^a^						Sick Child Care Facilities^b^						
	*N* Facilities offering Delivery Care	Injectable			Oral		*N* Facilities offering Sick Child Care	Injectable				Oral	
		Ampicillin	Procaine Penicillin	Gentamicin	Amoxicillin	Cotrimoxazole		Ampicillin	Penicillin	Ceftriaxone	Gentamicin	Amoxicillin	Cotrimoxazole
**AFRO**													
*Median*		*20%*	*62%*	*42%*	*91%*			*13%*	*84%*	*11%*	*34%*	*75%*	*79%*
*Range*		*4%–71%*		*17%–79%*	*74%–95%*	*74%–95%*		*3%–59%*	*79%–93%*	*4%–82%*	*27%–70%*	*16%–88%*	*14%–80%*
Kenya, 2010	207	4%	62%	79%	92% have oral antibiotics	92% have oral antibiotics	666	3% (ampicillin or cloxacillin)	83%	31%	70%	75%	79%
Rwanda, 2007	257	71%		60%	95% have oral antibiotics	95% have oral antibiotics	347	59% (ampicillin or cloxacillin)	85%	6%	51%	88% of facilities have cotrimoxazole, amoxicillin, and chloramphenicol	88% facilities have cotrimoxazole, amoxicillin, and chloramphenicol
Tanzania, 2006	451			17%	89% have oral antibiotics	89% have oral antibiotics	605	8% (ampicillin or cloxacillin)	93%	16%	27%	77%	80%
Uganda, 2007	261			40%	74% have oral antibiotics	74% have oral antibiotics	481	14%	81%	6%	31%	16%	14%
Namimbia, 2009	256			43%	96% have oral antibiotics^c^	96% have oral antibiotics^c^	347	12%	85%	82%	30%	97%	96%
Ghana, 2002	357	20%		39%	83%	83%	404	18%	79%	4%	36%	71%	71%
**SEARO**													
Bangladesh, 1999	718	14%		14%			693	35%				77%	88%
**EMRO**													
Egypt, 2004	559	55% have injectable antibiotics	55% have injectable antibiotics	55% have injectable antibiotics	84% have oral antibiotics	84% have oral antibiotics	552	31%	73%	2%	40%	65%	60%

a Facilities that provide normal delivery services during childbirth.

b Facilities that provide “curative care for sick children.”

c Defined as oral amoxicillin, augmentin, ampicillin, or cotrimoxazole.

AFRO, African Region; EMRO, Eastern Mediterranean Region; SEARO, Southeast Asian Region.

#### Antibiotic pricing and affordability

Based on pricing data from the WHO/HAI surveys, the costs and affordability of treatment for neonatal sepsis (ten-day course for 3 kg baby) were estimated (Table 6, WHO Regions; Table S2, National Pricing data; Table S3, Affordability data). The data were from years 2001 to 2013, and were not adjusted for inflation. The estimated costs for a treatment course of ampicillin in Africa ranged from US$2.34 (public sector) to US$4.02 (private sector), equivalent to 1–1.6 days of work for the lowest-level government laborer. Injectable gentamicin pricing was relatively low (<US$1) and affordable (<0.5 days of work) in most regions. Ceftriaxone was substantially more expensive across all regions, with private sector pricing uniformly much higher than public sector costs (reaching as high as US$40/course in Eastern Mediterranean and Western-Pacific Regions) and accordingly less affordable. In Africa, a course of ceftriaxone cost the equivalent of five days of work in the public sector, and almost 16 days in the private sector. A common finding was where specific antibiotics were free in the public sector, availability was low, and private sector availability and cost were high.

**Table 6:** WHO/HAI antibiotic pricing data for treatment of neonatal sepsis by WHO region.

WHO Region Sector	Parental						Oral			
	Ampicillin (1 g/vial)^a^		Gentamicin (40 mg/ml)^b^		Ceftriaxone (1 g/vial injection)^c^		Amoxicillin (250 mg capsule/tablet)^d^		Cotrimoxazole (40+200 mg/5 ml)^e^	
	Unit Price (vial)	Total Cost (US$)	Unit Price (ml)	Total Cost (US$)	Unit Price (Vial)	Total Cost (US$)	Unit Price (Capsule)	Total Cost (US$)	Unit Price (ml)	Total Cost (US$)
**AFR**										
Public	0.47 (0.03–0.81)	2.34	0.12 (0.05–0.2)	0.47	3.04 (0.9)	15.20	0.02 (0–9.05)	0.31	0.01 (0–0.02)	0.58
Private	0.8(0.27–1.01)	4.02	0.11 (0.03–0.31)	0.45	5.24 (0.21–24.12)	26.20	0.03 (0.03–0.07)	0.43	0.01 (0.01–0.04)	1.08
**AMR**										
Public	0.04	0.21	0	—	0 (0–1.77)	—	0.04 (0.036)	0.47	0 (0–0.02)	—
Private	0.74	3.68	2.93 (0.42–5.44)	11.72	3.78 (1.7–6.1)	18.90	0.25 (0.09–0.41)	3.24	0.02 (0.02–0.05)	1.58
**EMR**										
Public			0.28	1.10	0 (0–0.91)	—	0 (0–0.05)	—	0 (0–.01)	—
Private			0.28	1.12	7.9 (1.03–31.9)	39.50	0.12 (0.09–0.52)	0.16	0.02 (0.004–0.06)	1.41
**SEAR**										
Public			0	—	0.81 (0–1.41)	4.06	0.04 (0–0.04)	0.52	0.01 (0–0.01)	0.83
Private			0.1 (0.8–0.9)	0.34	1.59 (1.37–1.59)	7.94	0.05 (0.04–0.08)	0.69	0.01 (0.004–0.01)	0.50
**WPR**										
Public			0.07	0.26	0.55 (0–0.55)	2.77	0.04 (0–0.06)	0.39	0.01 (0–0.02)	0.83
Private			0.08	0.33	8.49 (0.42–9.59)	42.45	0.05 (0.04–0.08)	0.65	0.02 (0.01–0.003)	1.99

Total cost is estimated for the course of treating a 3 kg neonatal child for 10 days. For capsular formulation, the assumption was reconstitution in sterile water. For oral suspension 10% wastage was assumed.

For vials, a shelf life of 24 hours was assumed for ceftriaxone. The data were from 2001 to 2013, and were not adjusted for inflation.

a Data based on national surveys from: AFR (*n* = 4), AMR (*n* = 1).

b Data based on national surveys from: AFR (*n* = 8), AMR (*n* = 2), EMR (*n* = 9), SEAR (*n* = 4), WPR (*n* = 5).

c Data based on national surveys from: AFR (*n* = 15), AMR (*n* = 9), EMR (*n* = 13), SEAR (*n* = 4), WPR (*n* = 5).

d Data based on national surveys from: AFR (*n* = 15), AMR (*n* = 9), EMR (*n* = 13), SEAR (*n* = 3), WPR (*n* = 4).

e Data based on national surveys from: AFR (*n* = 0), AMR (*n* = 1), EMR (*n* = 0), SEAR (*n* = 0), WPR (*n* = 0).

AFR, Africa; AMR, Americas; EMR, Eastern Mediterranean; SEAR, Southeast Asia; WPR, Western Pacific.

Overall the cost of Amoxicillin capsules was low (<US$1/treatment course) and affordable (less than one work day) in all regions, though prices for suspension would be at least 150% higher. Cotrimoxazole suspension was also low cost and affordable, with generally higher markup in the private sector.

### Study Question 3. Non-prescription Use of Antibiotics

**Figure 7:**
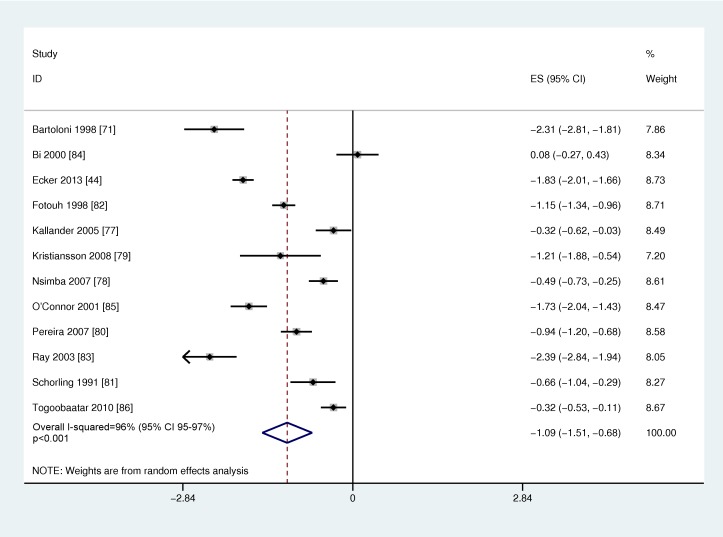
Meta-analysis of the logit of the prevalence of non-prescription over-the-counter antibiotic use by young infants and children in low- and middle-income countries. Effect size is the Logit (prevalence of antibiotic purchases that were obtained over the counter without a prescription).

Table 7 shows the study characteristics of the 12 studies reporting on non-prescription, over-the-counter use of antibiotics to treat illness in young children (<7 years of age). Six studies included infants; however, only one study separately reported use among the <1 year old infant age group [44]. One study reported on use <3 months of age and none on newborns [45]. Most of the studies were community-based cross-sectional surveys, while one study was an observation of pharmacy encounters. The conditions that were treated varied and included a wide range of illnesses (tonsillitis, respiratory illness, diarrhea) (Table 7). The overall quality of evidence on access was generally low (Table S1), clinically diverse, and statistically heterogeneous (overall I^2^ 96%, 95% CI 95%–97%). Random effects meta-analysis was conducted (Figure 7), and the global average of proportion of pediatric antibiotic purchases obtained over-the-counter was 25.1% (95% CI 18.1%–33.6%). Meta-regression showed no statistically significant difference based on WHO regional grouping.

**Table 7:** Over-the-counter access to antibiotics for children.

Author	Year	Country	WHO Region	Study Design	Study Setting	Rural/Urban	Child Age	Sample Size	Denominator	Percent Antibiotic Obtained Over-the-Counter
**Kallander [77]**	2005	Uganda	AFR	Cross-sectional	8 districts, community	Rural	<2 yo	328	Children with cough, difficulty breathing, and fever	42% of antibiotics in community obtained OTC
**Nsimba [78]**	2007	Tanzania	AFR	Pharmacy observation	Drug shops, rural Kibaha	Rural	<5 yo	279	Antibiotic purchases	38% not prescribed
**Bartoloni [71]**	1998	Bolivia	AMR	Cross-sectional	Community based household survey	Mixed	<72 months	188	Children receiving antibiotics in prior 4 months	9% from market
**Ecker [44]**	2013	Peru	AMR	Cross-sectional household survey	Community household surveys, Chorrillos, independencia, San Juan, Lima	Periurban	<5 yo	1,046	Children taking antibiotics	15.9% self-medication
**Kristiansson [79]**	2008	Peru	AMR	Cross-sectional household interviews	Rural community, Loreto	Rural	6–72 months	48	Antibiotics taken for pneumonia or dysentery	23% self prescribed
**Pereira [80]**	2007	Brazil	AMR	Cross-sectional survey	Community interviews, San Paulo	Urban	<2 yo	72	Amoxicillin users	6.4% self-prescribed
**Schorling [81]**	1991	Brazil	AMR	Prospective cohort	Illness surveillance project, Fortaleeza, Brazil	Urban	<5 yo	122	Children taking antibiotics	34% medicated by mothers or relatives
**Fotouh [82]**	1998	Egypt	EMR	Cross-sectional survey	Outpatient clinic, university hospital	NS	“Child”	577	Antibiotic purchases	24% self-prescription
**Ray [83]**	2003	India	SEAR	Cross-sectional household surveys	Community household surveys, Kolkata	Urban	Child (NS)	250	Antibiotic prescriptions	8.4% obtained over the counter
**Bi [84]**	2000	China	WPR	Cross-sectional survey	Students kindergarten, Hefei city	Urban	Kindergarten	125	Antibiotic purchases	52% self-medication
**O'Connor [85]**	2001	China	WPR	Cross-sectional survey, pharmacy observation	Kindergarten interviews, Beijing, Hebei	Mixed	3–6 yo	329	Antibiotics purchases	15% not prescribed by doctor or pharmacist
**Togoobaatar [86]**	2010	Mongolia	WPR	Cross-sectional household survey	Community based random sample 10 subdistricts capital city, Ulaanbaatar	Urban	<5 yo	356	Antibiotic purchases	42% self-medication

AFR, WHO African Region; AMR, Region of the Americas; EMR, Eastern Mediterranean Region; EUR, European Region; SEAR, Southeast Asia Region; WPR, Western Pacific Region.

While there were few studies that reported specifically on infants, several studies indicated that non-prescription use may be lower in the younger ages. In Peru, among infants <12 months, self-medication rates were lower at 7.6% compared to 15.9% in the entire under-five population. Similarly in Brazil, rates of non-prescription use were 6.5% among children <2 years old compared to 28.1% for children 2–7 years of age. In the single study that reported on self-medication among infants <3 months of age with illness, none of the children in this age group had taken a non-prescription antibiotic [45].

## Discussion

In this landscape review, we found that trained frontline health workers may use clinical sign algorithms to detect pBI in young infants with relatively high sensitivity in certain settings, but lower specificity. Availability of first-line injectable antibiotics to treat neonatal infections was low in first-level health facilities in Southeast Asia and Africa, and data on neonatal-specific formulations were limited. Oral antibiotics were generally highly available and affordable. The procurement of antibiotics over-the-counter without a prescription was common in developing countries in children under five, accounting for one in four antibiotic purchases; while non-prescription use in young infants may be lower, this needs to be evaluated with respect to safety and development of antimicrobial resistance.

### Frontline Health Worker Diagnosis of Severe Disease/pBI in Young Infants

The development, refinement, and simplification of IMCI algorithms to identify sick young infants have been a major advancement to increasing diagnosis of and access to treatment for neonatal infections in LMICs. These analyses provide evidence that clinical-sign-based algorithms can detect neonatal infection with relatively high sensitivity, and that frontline health workers can use these algorithms to identify pBI with relatively good sensitivity and lower specificity compared to physician diagnosis. These data are promising for the 50 million annual home births and many sick newborns who first present to peripheral facilities.

However, there are also several limitations to these data. Most of the facility-based validation studies were at risk for selection bias, given that the assessment was among children for whom parents sought care for illness. They may have been sicker or presented at facilities with better trained staff. Given this initial selection, the sensitivity and specificity of the diagnosis may not reflect the performance of population-based screening. Studies to determine the sensitivity, specificity, and predictive value of the algorithms are needed at the population/community level, and would also be useful to examine by morbidity pattern. Some studies were from neonatal research studies where quality of training and care may differ from routine health delivery systems in LMICs, and the health worker may be aware of their being assessed. In the larger scale program evaluations of IMNCI in Purilia, India, only one-third of CHWs actually completed the full seven-sign assessment and the agreement was much lower than the data from neonatal research trials. Thus, the performance reflected in the meta-analysis may reflect an optimal performance and potentially overrate a programmatic setting. Other limitations to this analysis included that the clinical sign algorithms varied between evaluations, and different algorithms may have varying diagnostic accuracy. The algorithms used were either the IMCI algorithm at the time of the original study, or the “best” performing algorithm chosen by the authors within the particular validation studies. Furthermore, preterm infants may share some clinical signs as newborns with infection, and also carry higher risk of infection. Thus understanding the validity of these algorithms in full term versus preterm babies is important; however, none of the studies provided data to examine this question. Another important consideration is that the majority of the validation studies included in the meta-analysis were conducted in South Asia. In the Young Infant Clinical Signs group study, the algorithm had lower sensitivity in the two African sites, and the potential influence of HIV infection was raised by the authors. Thus, these findings may be more generalizable to similar Asian settings, and further evaluation is needed in the African setting. Finally, the specificity of sign-based algorithms was relatively low, particularly when using a laboratory-based gold standard. The negative impact of over-referral and overtreatment, including burdening already strained health systems and enhancement of antibiotic resistance, remain major challenges. These data emphasize the potential impact of novel technologies such as biomarkers and low-cost point of care testing to facilitate the diagnostic process and improve accuracy of detection of newborn infections.

### Antibiotic Availability

The UN Commission for Life-Saving Commodities for Women and Children has prioritized increasing access to injectable antibiotics for neonatal sepsis as a key commodity. In our review, we found that first-line injectable antibiotics to treat neonatal infections have relatively low and variable availability in Africa and Southeast Asia, where the majority of global neonatal and child deaths and infections occur. Injectable first-line agents for treating neonatal infections (ampicillin and gentamicin) had low-to-moderate availability in outpatient child health or delivery facilities in Africa and Southeast Asia, and there were scarce data on procaine benzylpenicillin (which is low cost and allows once daily intramuscular dosing). Ceftriaxone, a second-line regimen, also had low availability particularly in Africa in both SPA and HAI surveys, with discrepant availability in the public versus private sector. Overall the evidence on antibiotic availability was of moderate quality. While the data quality in the literature was low (primarily sub-national and small sample size), both SPA and HAI data were nationally representative data, with random sampling, large samples sizes, and standardized methodology. However, data on neonatal formulations and concentrations were generally scarce, particularly when analyzed by region. There were no data on the lower concentrations of gentamicin (10–20 mg/ml) or smaller doses for reconstitution of ceftriaxone (250 mg vials), which are easier to administer in neonates [21]. Future efforts need to routinely collect data in standardized surveys regarding these essential neonatal medications and formulations, which are now on the WHO Model List of Essential Medicines for Children.

### Antibiotic Pricing and Affordability

Antibiotic pricing and affordability are critical determinants of access for the poor. The UN Millennium Development Target 17 specifically aims “in cooperation with pharmaceutical companies, to provide access to affordable, essential drugs in developing countries.” Generic brands were typically lower cost than originator brands, and prices were substantially lower or often free in the public sector, however availability was often low. An injectable course of gentamicin for treating neonatal sepsis was low cost in most regions (<US$1), with slightly higher cost for ampicillin (US$2–4 in Africa). However, the cost of ceftriaxone was very high, ranging from ∼US$4–US$8 per treatment course in the public sector in Southeast Asia to over US$30 in the African public sector. The high cost and low affordability of ceftriaxone are a major barrier to access for the poor; for example in Africa, its purchase required a median 16 days of working wages for an unskilled government employee. A recent report to the UN Commission on Live-Saving Commodities called for further investigation into the common supply and manufacturing sources and national regulatory and financing processes to better understand the bottlenecks to procurement [21]. Concerted efforts by pharmaceutical and government agencies need to be made to increase supply and availability of these essential antibiotics, particularly neonatal formulations, and reduce costs to consumers (by increasing government subsidies or coverage of these key medications under insurance schemes) and duties and taxes placed on these medicines, to improve the affordability and access.

### Special Considerations for Antibiotic Formulations and Delivery for Neonates in LMICs

Specific consideration must be given to antibiotic formulations and delivery mechanisms for neonates and young children in the developing country setting [46],[47]. Darmstadt and colleagues have identified several key issues including the availability of extended interval dosing (>24 hours), safety and efficacy of intramuscular dosing, clearance mechanisms given the inability to closely monitor laboratory tests and fluid status, supply logistics and storage, ease of dilution and administration, drug stability, availability of multiple versus single use vials, availability of oral suspension/syrup, and variable gastrointestinal absorption of oral antibiotics in newborns and during illness [22],[27]. A recent case study for the UN Commission on Life-Saving Commodities identified several challenges to availability and use of injectable antibiotics in LMICs [21],[48]. Providing intravenous or frequent dosing antibiotics is difficult in low-income countries where human resources are limited and the placement of neonatal and pediatric IV catheters requires special skills and training. In three African SPAs, intravenous catheters were only available in 27% of first-level facilities. Intramuscular injection may therefore be the preferred delivery mechanism in the community and first-level facilities, and novel mechanisms have been tested for delivery, including Uniject and Microneedle patch [21]. Ceftriaxone powder must be reconstituted in sterile water and may only be stored for 24 hours afterwards, thus 1 g vials may lead to waste in low-volume or acuity facilities. Gentamicin requires close monitoring given risk of renal and ototoxicity; however, this may often not be possible in LMIC settings. Future work should assess safety of specific neonatal formulations and challenges/barriers to administration, storage, and supply logistics.

While treatment with injectable antibiotics is standard of care for treating serious neonatal infections in high-income settings, feasibility may be limited in developing countries where availability and administration are challenges within weak health systems, and simplified regimens including oral antibiotics may be preferable to none [27]. A meta-analysis showed that community-based case management of neonatal pneumonia may result in significant reductions in neonatal (27%, 95% CI 18%–35%) and pneumonia-specific mortality (42%, 95% CI 22%–57%); four of the six included trials used oral antibiotics. The recently completed Simplified Antibiotic Therapy Trials in Bangladesh, Pakistan, Democratic Republic of Congo, Kenya, and Nigeria will compare the efficacy of simpler regimens to treat neonatal sepsis utilizing less frequent injections and oral amoxicillin [49]–[51]. In the SEARCH trial conducted in Gadichiroli, India, cotrimoxazole in combination with gentamicin [27] was highly effective in reducing neonatal sepsis case fatality by 60%. In a later community-based study in Karachi, Pakistan treatment failure with cotrimoxazole-gentamicin was significantly higher than penicillin-gentamicin [17]. Amoxicillin and cotrimoxazole were both highly available and low cost, and could be administered by parents. Thus, administration of oral antibiotics, if demonstrated to have equivalent effectiveness, may hold promise for increasing access to treatment of pSBI in LMICs.

### Non-prescription Antibiotic Use

This work confirms that informal and over-the-counter mechanisms for obtaining antibiotics are a substantial market in developing countries and may account for up to 25% of antibiotic purchases for children under five in LMICs. The lack of data on neonatal use and the diversity of conditions being treated were limitations. The limited data indicate that use may be lower among young infants than children. However, these data highlight a critical area for future work and the importance of monitoring appropriate antibiotic use in developing countries. Newborns have special considerations regarding dosing, metabolism, and adverse effects that require closer monitoring than older children, and render unmonitored antibiotic use more hazardous. Furthermore, rates of counterfeit medications are high in LMICs, with a median prevalence of 29% [52].

### Antibiotic Resistance

The emergence of antibiotic resistance as a result of inappropriate antibiotic use is a huge, emerging concern in LMICs, and has been recently highlighted in the WHO Global Report on Surveillance of Antimicrobial Resistance [53]. Our work has identified potential areas of antibiotic overuse in LMICs, both from parental self-medication without prescriptions, as well as the health workers' use of sign-based algorithms with low specificity. Zaidi and colleagues previously reviewed data on antibiotic resistance among hospital-acquired pathogens in LMICs and reported alarmingly high rates of resistance—over 50% of *E. coli* and *Klebsiella* were resistant to gentamicin and >40% to cefotaxime [8]. Multidrug resistance is becoming more common (75% of gram negative organisms in Africa [54]) as well as resistance to second- and third-line antimicrobial agents (for example with *Klebsiella* 51% cefotaxime resistant, and 37% amikacin resistant) [8]. One study in a developing country neonatal intensive care unit showed that using 3rd generation cephalosporins as first-line treatment for sepsis was associated with 18-fold increased risk of the development of antibiotic resistance [55]. In a recent review of community-acquired neonatal pathogens, the most common pathogens (*S. aureus*, *E. coli*, and *Klebsiella*) were >40% resistant (or had reduced sensitivity) to the combination of penicillin and gentamicin or 3rd generation cephalosporins [18]. In particular, *Klebsiella* was not susceptible (at >50%) to any antibiotic tested (penicillin, gentamicin, chloramphenicol, or 3rd generation cephalosporins) [18]. In the WHO global surveillance report, high rates of resistance (>50%) of *E. coli* and *Klebsiella* to 3rd generation cephalosporins as well as methicillin-resistant *S. aureus* (MRSA) have now been widely reported in almost all WHO regions [53]. The emergence of antimicrobial resistance will reduce the efficacy of treatment and narrow the armamentarium of available medications for treating neonatal sepsis in the future generations. Zaidi and colleagues suggest that 70% of hospital-acquired neonatal infections in low-resource settings may not be covered by empiric typical first-line regimens for treating neonatal sepsis (ampicillin and gentamicin) [8], and a recent review of community-acquired bacteremia has also questioned the efficacy and appropriateness of WHO's current recommended antibiotics for neonatal sepsis [18], particularly for second-line therapy given the risk of resistance propagation with third-generation cephalosporins. There is even more limited data available regarding the availability of medications for resistant, nosocomial infections. For example in our review, no data were available on carbapenem and one article cited that colistin was not procured [56]. Routine data and surveillance on microbial etiology and resistance patterns in LMICs are required in order to properly target treatment guidelines.

### Hospital Acquired Infections

Although not a focus of the current review, when addressing neonatal infections in LMICs, it is critical to consider the specific challenges of hospital-acquired infections. Rates of neonatal infections among hospital-born babies in LMICs may be 3–20 times higher than rates in high-income settings [8], in large part due to unhygienic practices during labor, delivery, and the postnatal period. In the Indian National Neonatal-Perinatal Database, the rate of blood-culture confirmed sepsis was 15.6/1,000 live births in 16 national referral level nurseries [57], while in a Nigerian report, blood stream infections affected 6.8% of low birth weight babies [58]. Pathogens associated with hospital-acquired neonatal infections are different in low-resource settings, where over 60% may be due to gram negative rods (*Klebsiella pneumonia*, *E. coli*, P*seudomonas*, and *Acinetobacter*), which proliferate in multi-use containers of soaps and solutions [8], and *S. aureus* is also common. Prolonged therapy, empiric use of broad spectrum antibiotics, and unhygienic practices and environments propagate the selection and rapid spread of these highly resistant nosocomial pathogens in LMIC facilities [8].

### Study Limitations

A major limitation of this analysis was the scarcity of neonatal-specific data, and thus to answer some of our study questions, we expanded the age range of our population to include young infants and children under five. Some of the summary statistics represented few surveys for a region, and/or we made assumptions regarding higher concentrations or different formulations to project to neonates. The pricing data were not adjusted for inflation. Given the landscape nature of the review, the scope of the review was broad and it is possible that the individual searches or search terms may have missed articles. However, we conducted separate detailed searches, including multiple search terms for each of the three original research questions detailed in the web appendix, and searched a wide range of databases, unpublished gray literature sources and donor websites, and bibliographies of sentinel articles. Our grey literature searches however were limited, as we did not consult existing programs, researchers, or governments. This outreach should be pursued in future work, some of which is currently being addressed in the Every Newborn Action Plan. Future efforts also need to be made to routinely collect data on WHO Essential Medications for Children in standardized surveys (SPAs, WHO/HAI), specifically on neonatal formulations (lower concentrations and procaine benzylpenicillin) as well as to assess the quality and validity of IMNCI evaluations in young infants in SPAs.

### Conclusions

Improving diagnosis and access to treatment for neonatal infections are critical steps to reducing neonatal morbidity and mortality. Frontline health workers may be trained to accurately detect pBI, but ensuring adequate quality of program implementation remains a challenge in large-scale programs. The availability of injectable agents to treat neonatal sepsis was generally low with few data on neonatal formulations. Furthermore, over-the-counter mechanisms for obtaining antibiotics were common and needs improved monitoring and regulation in order to avert the propagation of antibiotic resistance. The development of novel, low-cost, and user-friendly diagnostics to improve the accuracy of detecting neonatal infections may play a critical role in improving access to treatment and reducing inappropriate antibiotic use in low-resource settings. Concerted efforts by governments, policymakers, and the pharmaceutical industry are needed to improve the availability and pricing of life-saving antimicrobial agents in LMICs.

## Supporting Information

Figure S1 QUADAS-2 quality assessment summary graphs.
(DOC)

Table S1 Evidence tables and individual quality assessment for all research questions.
(XLSX)

Table S2 WHO/HAI Database: Antibiotic Availability and Pricing National Data.
(XLSX)

Table S3 WHO affordability data.
(XLSX)

Text S1 PRISMA statement.
(DOC)

Text S2 Search strategy and terms.
(DOC)

Text S3 Research protocol.
(DOC)

## Abbreviations

CHW: community health worker
HAI: Health Action International
IMCI: Integrated management of Childhood Illness
IMNCI: Integrated Management of Neonatal and Childhood Illnesses
LMIC: low- or middle-income country
pBI: possible bacterial infection
pSBI: possible severe bacterial infection
SPA: service provision assessment
VSD: very severe disease
PVSD: possible very severe disease
