# Supplementary material for: Treatment of Infections in Young Infants in Low- and Middle-Income Countries: A Systematic Review and Meta-analysis of Frontline Health Worker Diagnosis and Antibiotic Access
Source: PLoS Med. 2014 Oct 14;11(10):e1001741. doi: 10.1371/journal.pmed.1001741 (PMC4196753; doi:10.1371/journal.pmed.1001741)

**Figure S1**

**Quality assessment summary for studies of health worker diagnosis of pSBI**

QUADAS-2 Assessment of Risk of Bias


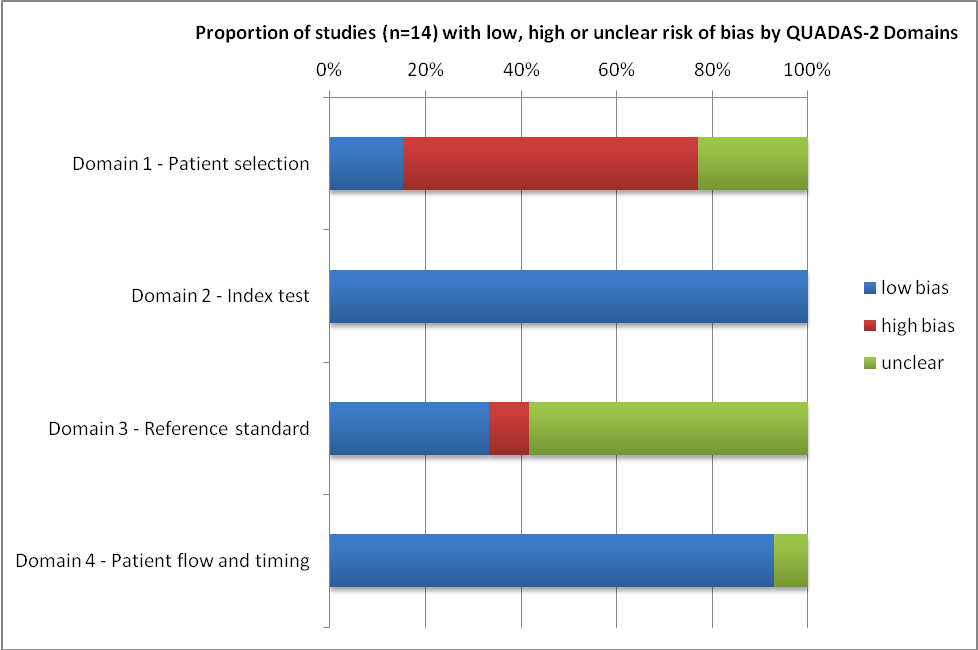


QUADAS-2 Assessment of Applicability Concerns


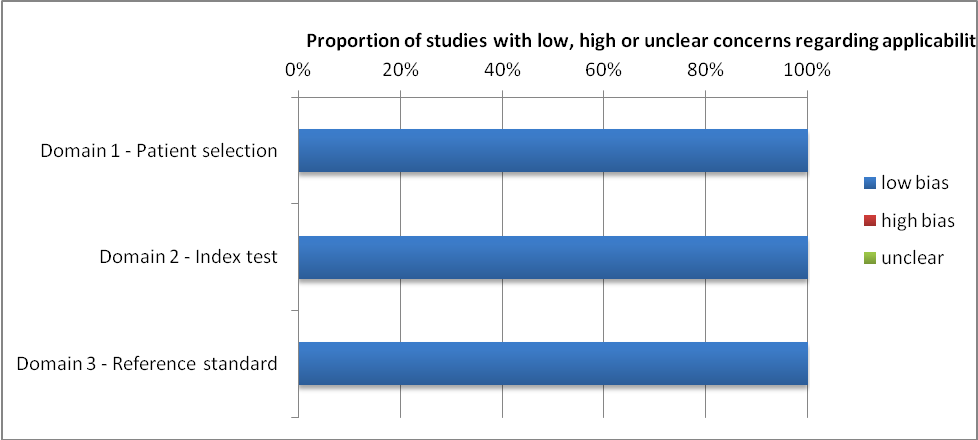

Supplement: Figure S1 — QUADAS-2 quality assessment summary graphs. (DOC) [file pmed.1001741.s001.doc]
