# Supplementary material for: Treatment of Infections in Young Infants in Low- and Middle-Income Countries: A Systematic Review and Meta-analysis of Frontline Health Worker Diagnosis and Antibiotic Access
Source: PLoS Med. 2014 Oct 14;11(10):e1001741. doi: 10.1371/journal.pmed.1001741 (PMC4196753; doi:10.1371/journal.pmed.1001741)
Supplement: Text S2 — Search strategy and terms. (DOC) [file pmed.1001741.s006.doc]

**SEARCH TERMS FOR NEONATAL ANTIBIOTIC ACCESS REVIEW – PUBLISHED LITERATURE**

**Research Question 1 (Original Searches February 2010, updated June 2013, updated May 2014)**

***1) What proportion of newborns with possible severe bacterial infection (PSBI) are recognized and prescribed antibiotics by a health provider?***

***Strategy 1: Search terms for IMCI***

*IMCI[All Fields] OR IMNCI[All Fields] OR integrated[All Fields] AND ("organization and administration"[MeSH Terms] OR ("organization"[All Fields] AND "administration"[All Fields]) OR "organization and administration"[All Fields] OR "management"[All Fields] OR "disease management"[MeSH Terms] OR ("disease"[All Fields] AND "management"[All Fields]) OR "disease management"[All Fields] OR ('possible severe bacterial infection'[all]) OR ('possible serious bacterial infection'[all])) AND ("Childhood"[Journal] OR "childhood"[All Fields]) AND illness[All Fields]*

***Strategy 2: Combination of search terms for “neonatal/infant” & “algorithm” & “infections” & “developing country” terms***

***Search terms of ‘neonatal / infant’***

((infant[MeSH] OR (infant[MeSH] OR child[MeSH] OR adolescent[MeSH]))) AND (("infant, newborn"[MeSH Terms] OR ("infant"[All Fields] AND "newborn"[All Fields]) OR "newborn infant"[All Fields] OR "neonatal"[All Fields]) OR (neonat* OR newborn*))

***Search terms for ‘algorithm’***

*(algorithm[All Fields] OR "child health services"[MeSH] OR "community health workers"[MeSH] OR "risk assessment"[MeSH] OR algorithm OR "community health workers"[All Fields] OR "health volunteers"[All Fields] OR "health workers"[All Fields] OR "frontline" OR "front line" OR "risk assessment"[All Fields])*

***Search terms for ‘ infections’***

*((("umbilical cord"[MeSH Terms] OR ("umbilical"[All Fields] AND "cord"[All Fields]) OR "umbilical cord"[All Fields])) AND (Infections[All Fields] AND "infection"[MeSH Terms] OR "infection"[All Fields] OR "communicable diseases"[MeSH Terms] OR ("communicable"[All Fields] AND "diseases"[All Fields]) OR "communicable diseases"[All Fields])) OR (((Neonate AND "infant, newborn"[MeSH Terms] OR ("infant"[All Fields] AND "newborn"[All Fields]) OR "newborn infant"[All Fields] OR "neonatal"[All Fields])) AND (Cellulitis : "cellulitis"[MeSH Terms] OR "cellulitis"[All Fields])) OR (((Neonate AND "infant, newborn"[MeSH Terms] OR ("infant"[All Fields] AND "newborn"[All Fields]) OR "newborn infant"[All Fields] OR "neonatal"[All Fields])) AND ((Fever : "fever"[MeSH Terms] OR "fever"[All Fields]) OR (Pyrexia : "fever"[MeSH Terms] OR "fever"[All Fields] OR "pyrexia"[All Fields]))) OR (((Neonate AND "infant, newborn"[MeSH Terms] OR ("infant"[All Fields] AND "newborn"[All Fields]) OR "newborn infant"[All Fields] OR "neonatal"[All Fields])) AND (Bacteremia : "bacteraemia"[All Fields] OR "bacteremia"[MeSH Terms] OR "bacteremia"[All Fields])) OR (((Neonate AND "infant, newborn"[MeSH Terms] OR ("infant"[All Fields] AND "newborn"[All Fields]) OR "newborn infant"[All Fields] OR "neonatal"[All Fields])) AND (Diarrhea : "diarrhoea"[All Fields] OR "diarrhea"[MeSH Terms] OR "diarrhea"[All Fields])) OR (((Neonate AND "infant, newborn"[MeSH Terms] OR ("infant"[All Fields] AND "newborn"[All Fields]) OR "newborn infant"[All Fields] OR "neonatal"[All Fields])) AND (Acute lower Respiratory Infection : acute[All Fields] AND lower[All Fields] AND ("respiratory tract infections"[MeSH Terms] OR ("respiratory"[All Fields] AND "tract"[All Fields] AND "infections"[All Fields]) OR "respiratory tract infections"[All Fields] OR ("respiratory"[All Fields] AND "infection"[All Fields]) OR "respiratory infection"[All Fields]))) OR (((Neonate AND "infant, newborn"[MeSH Terms] OR ("infant"[All Fields] AND "newborn"[All Fields]) OR "newborn infant"[All Fields] OR "neonatal"[All Fields])) AND (Tetanus AND "tetanus"[MeSH Terms] OR "tetanus"[All Fields])) OR (((Neonate AND "infant, newborn"[MeSH Terms] OR ("infant"[All Fields] AND "newborn"[All Fields]) OR "newborn infant"[All Fields] OR "neonatal"[All Fields])) AND (Urinary Tract Infection AND "urinary tract infections"[MeSH Terms] OR ("urinary"[All Fields] AND "tract"[All Fields] AND "infections"[All Fields]) OR "urinary tract infections"[All Fields] OR ("urinary"[All Fields] AND "tract"[All Fields] AND "infection"[All Fields]) OR "urinary tract infection"[All Fields])) OR (((Neonate AND "infant, newborn"[MeSH Terms] OR ("infant"[All Fields] AND "newborn"[All Fields]) OR "newborn infant"[All Fields] OR "neonatal"[All Fields])) AND (Pneumonia AND "pneumonia"[MeSH Terms] OR "pneumonia"[All Fields] AND lower[All Fields] AND ("respiratory tract infections"[MeSH Terms] OR ("respiratory"[All Fields] AND "tract"[All Fields] AND "infections"[All Fields]) OR "respiratory tract infections"[All Fields] OR ("respiratory"[All Fields] AND "tract"[All Fields] AND "infection"[All Fields]) OR "respiratory tract infection"[All Fields]))) OR (((Neonate AND "infant, newborn"[MeSH Terms] OR ("infant"[All Fields] AND "newborn"[All Fields]) OR "newborn infant"[All Fields] OR "neonatal"[All Fields])) AND (Meningitis AND "meningitis"[MeSH Terms] OR "meningitis"[All Fields])) OR (((Neonate AND "infant, newborn"[MeSH Terms] OR ("infant"[All Fields] AND "newborn"[All Fields]) OR "newborn infant"[All Fields] OR "neonatal"[All Fields])) AND (Sepsis[All Fields] AND "septicaemia"[All Fields] OR "sepsis"[MeSH Terms] OR "sepsis"[All Fields] OR "septicemia"[All Fields] OR "Septicemia"[All Fields])) OR (((Neonate AND "infant, newborn"[MeSH Terms] OR ("infant"[All Fields] AND "newborn"[All Fields]) OR "newborn infant"[All Fields] OR "neonatal"[All Fields])) AND (Infections[All Fields] AND "infection"[MeSH Terms] OR "infection"[All Fields] OR "communicable diseases"[MeSH Terms] OR ("communicable"[All Fields] AND "diseases"[All Fields]) OR "communicable diseases"[All Fields])) OR ((("umbilical cord"[MeSH Terms] OR ("umbilical"[All Fields] AND "cord"[All Fields]) OR "umbilical cord"[All Fields])) AND (Infections[All Fields] AND "infection"[MeSH Terms] OR "infection"[All Fields] OR "communicable diseases"[MeSH Terms] OR ("communicable"[All Fields] AND "diseases"[All Fields]) OR "communicable diseases"[All Fields]))*

***Search terms for developing countries***

((("Cote d'Ivoire"[All Fields] OR "eritrea"[MeSH Terms] OR ("eritrea"[MeSH Terms] OR "eritrea"[All Fields]) OR "ethiopia"[MeSH Terms] OR ("ethiopia"[MeSH Terms] OR "ethiopia"[All Fields]) OR "gambia"[MeSH Terms] OR ("gambia"[MeSH Terms] OR "gambia"[All Fields]) OR "ghana"[MeSH Terms] OR ("ghana"[MeSH Terms] OR "ghana"[All Fields]) OR "guinea"[MeSH Terms] OR ("guinea"[MeSH Terms] OR "guinea"[All Fields]) OR "guinea-bissau"[MeSH Terms] OR ("guinea-bissau"[MeSH Terms] OR "guinea-bissau"[All Fields] OR ("guinea"[All Fields] AND "bissau"[All Fields]) OR "guinea bissau"[All Fields]) OR "haiti"[MeSH Terms] OR ("haiti"[MeSH Terms] OR "haiti"[All Fields]) OR "india"[MeSH Terms] OR ("india"[MeSH Terms] OR "india"[All Fields]) OR "kenya"[MeSH Terms] OR ("kenya"[MeSH Terms] OR "kenya"[All Fields]) OR Democratic[All Fields] AND ("korea"[All Fields] OR "korea"[MeSH Terms]) OR "Kyrgyz Republic"[All Fields] OR "Lao PDR"[All Fields] OR ("lesotho"[MeSH Terms] OR "lesotho"[All Fields]) OR "liberia"[MeSH Terms] OR ("liberia"[MeSH Terms] OR "liberia"[All Fields]) OR ("madagascar"[MeSH Terms] OR "madagascar"[All Fields]) OR "malawi"[MeSH Terms] OR ("malawi"[MeSH Terms] OR "malawi"[All Fields]) OR "mali"[MeSH Terms] OR ("mali"[MeSH Terms] OR "mali"[All Fields]) OR "mauritania"[MeSH Terms] OR ("mauritania"[MeSH Terms] OR "mauritania"[All Fields]) OR "moldova"[MeSH Terms] OR ("moldova"[MeSH Terms] OR "moldova"[All Fields]) OR "mongolia"[MeSH Terms] OR ("mongolia"[MeSH Terms] OR "mongolia"[All Fields]) OR "mozambique"[MeSH Terms] OR ("mozambique"[MeSH Terms] OR "mozambique"[All Fields]) OR "myanmar"[MeSH Terms] OR ("myanmar"[MeSH Terms] OR "myanmar"[All Fields]) OR "nepal"[MeSH Terms] OR ("nepal"[MeSH Terms] OR "nepal"[All Fields]) OR "nicaragua"[MeSH Terms] OR ("nicaragua"[MeSH Terms] OR "nicaragua"[All Fields]) OR "niger"[MeSH Terms] OR ("niger"[MeSH Terms] OR "niger"[All Fields]) OR "nigeria"[MeSH Terms] OR ("nigeria"[MeSH Terms] OR "nigeria"[All Fields]) OR "North Korea"[All Fields] OR "DPRK"[All Fields] OR "pakistan"[MeSH Terms] OR ("pakistan"[MeSH Terms] OR "pakistan"[All Fields]) OR "Papua New Guinea"[All Fields] OR "rwanda"[MeSH Terms] OR ("rwanda"[MeSH Terms] OR "rwanda"[All Fields]) OR "Sao Tome and Principe"[All Fields] OR "senegal"[MeSH Terms] OR ("senegal"[MeSH Terms] OR "senegal"[All Fields]) OR "Sierra Leone"[All Fields] OR "Solomon Islands"[All Fields] OR "somalia"[MeSH Terms] OR ("somalia"[MeSH Terms] OR "somalia"[All Fields]) OR "sudan"[MeSH Terms] OR ("sudan"[MeSH Terms] OR "sudan"[All Fields]) OR "tajikistan"[MeSH Terms] OR ("tajikistan"[MeSH Terms] OR "tajikistan"[All Fields]) OR "tanzania"[MeSH Terms] OR ("tanzania"[MeSH Terms] OR "tanzania"[All Fields]) OR "east timor"[All Fields] OR "east timor"[MeSH Terms] OR ("east timor"[MeSH Terms] OR ("east"[All Fields] AND "timor"[All Fields]) OR "east timor"[All Fields] OR ("timor"[All Fields] AND "leste"[All Fields]) OR "timor leste"[All Fields]) OR "togo"[MeSH Terms] OR ("togo"[MeSH Terms] OR "togo"[All Fields]) OR "uganda"[MeSH Terms] OR ("uganda"[MeSH Terms] OR "uganda"[All Fields]) OR "uzbekistan"[MeSH Terms] OR ("uzbekistan"[MeSH Terms] OR "uzbekistan"[All Fields]) OR "vietnam"[MeSH Terms] OR ("vietnam"[MeSH Terms] OR "vietnam"[All Fields]) OR "yemen"[MeSH Terms] OR ("yemen"[MeSH Terms] OR "yemen"[All Fields]) OR "Republic of Yemen"[All Fields] OR "democratic republic of the congo"[All Fields] OR "democratic republic of the congo"[MeSH Terms] OR ("democratic republic of the congo"[MeSH Terms] OR ("democratic"[All Fields] AND "republic"[All Fields] AND "congo"[All Fields]) OR "democratic republic of the congo"[All Fields] OR "zaire"[All Fields]) OR "zambia"[MeSH Terms] OR ("zambia"[MeSH Terms] OR "zambia"[All Fields]) OR "zimbabwe"[MeSH Terms] OR ("zimbabwe"[MeSH Terms] OR "zimbabwe"[All Fields]) OR "albania"[MeSH Terms] OR ("albania"[MeSH Terms] OR "albania"[All Fields]) OR "algeria"[MeSH Terms] OR ("algeria"[MeSH Terms] OR "algeria"[All Fields]) OR "angola"[MeSH Terms] OR ("angola"[MeSH Terms] OR "angola"[All Fields]) OR "armenia"[MeSH Terms] OR ("armenia"[MeSH Terms] OR "armenia"[All Fields]) OR "azerbaijan"[MeSH Terms] OR ("azerbaijan"[MeSH Terms] OR "azerbaijan"[All Fields]) OR "byelarus"[MeSH Terms] OR ("byelarus"[MeSH Terms] OR "byelarus"[All Fields] OR "belarus"[All Fields]) OR "bolivia"[MeSH Terms] OR ("bolivia"[MeSH Terms] OR "bolivia"[All Fields]) OR "Bosnia and Herzegovina"[All Fields] OR "brazil"[MeSH Terms] OR ("brazil"[MeSH Terms] OR "brazil"[All Fields]) OR "bulgaria"[MeSH Terms] OR ("bulgaria"[MeSH Terms] OR "bulgaria"[All Fields]) OR "Cape Verde"[All Fields] OR "china"[MeSH Terms] OR ("china"[MeSH Terms] OR "china"[All Fields]) OR "colombia"[MeSH Terms] OR ("colombia"[MeSH Terms] OR "colombia"[All Fields]) OR "cuba"[MeSH Terms] OR ("cuba"[MeSH Terms] OR "cuba"[All Fields]) OR "djibouti"[MeSH Terms] OR ("djibouti"[MeSH Terms] OR "djibouti"[All Fields]) OR "Dominican Republic"[All Fields] OR "ecuador"[MeSH Terms] OR ("ecuador"[MeSH Terms] OR "ecuador"[All Fields]) OR "egypt"[MeSH Terms] OR ("egypt"[MeSH Terms] OR "egypt"[All Fields]) OR "Arab Republic of Egypt"[All Fields] OR "El Salvador"[All Fields] OR "fiji"[MeSH Terms] OR ("fiji"[MeSH Terms] OR "fiji"[All Fields]) OR "georgia republic"[MeSH Terms] OR "georgia"[tiab] OR "georgia (republic)"[MeSH Terms] OR "guatemala"[MeSH Terms] OR ("guatemala"[MeSH Terms] OR "guatemala"[All Fields]) OR "guyana"[MeSH Terms] OR ("guyana"[MeSH Terms] OR "guyana"[All Fields]) OR "honduras"[MeSH Terms] OR ("honduras"[MeSH Terms] OR "honduras"[All Fields]) OR "indonesia"[MeSH Terms] OR ("indonesia"[MeSH Terms] OR "indonesia"[All Fields]) OR "iran"[MeSH Terms] OR ("iran"[MeSH Terms] OR "iran"[All Fields]) OR "Islamic Republic of Iran"[All Fields] OR "iraq"[MeSH Terms] OR ("iraq"[MeSH Terms] OR "iraq"[All Fields]) OR "jamaica"[MeSH Terms] OR ("jamaica"[MeSH Terms] OR "jamaica"[All Fields]) OR "jordan"[MeSH Terms] OR ("jordan"[MeSH Terms] OR "jordan"[All Fields]) OR "kazakhstan"[MeSH Terms] OR ("kazakhstan"[MeSH Terms] OR "kazakhstan"[All Fields]) OR "micronesia"[All Fields] OR "micronesia"[MeSH Terms] OR ("micronesia"[MeSH Terms] OR "micronesia"[All Fields] OR "kiribati"[All Fields]) OR "macedonia republic"[MeSH Terms] OR ("macedonia (republic)"[MeSH Terms] OR ("macedonia"[All Fields] AND "(republic)"[All Fields]) OR "macedonia (republic)"[All Fields] OR "macedonia"[All Fields]) OR "FYR of Macedonia"[All Fields] OR "Former Yugoslav Republic of Macedonia"[All Fields] OR "indian ocean islands"[All Fields] OR "indian ocean islands"[MeSH Terms] OR ("indian ocean islands"[MeSH Terms] OR ("indian"[All Fields] AND "ocean"[All Fields] AND "islands"[All Fields]) OR "indian ocean islands"[All Fields] OR "maldives"[All Fields]) OR "Marshall Islands"[All Fields] OR "micronesia"[MeSH Terms] OR ("micronesia"[MeSH Terms] OR "micronesia"[All Fields]) OR "Federated States of Micronesia"[All Fields] OR "morocco"[MeSH Terms] OR ("morocco"[MeSH Terms] OR "morocco"[All Fields]) OR "namibia"[MeSH Terms] OR ("namibia"[MeSH Terms] OR "namibia"[All Fields]) OR "paraguay"[MeSH Terms] OR ("paraguay"[MeSH Terms] OR "paraguay"[All Fields]) OR "peru"[MeSH Terms] OR ("peru"[MeSH Terms] OR "peru"[All Fields]) OR "philippines"[MeSH Terms] OR ("philippines"[MeSH Terms] OR "philippines"[All Fields]) OR "romania"[MeSH Terms] OR ("romania"[MeSH Terms] OR "romania"[All Fields]) OR "samoa"[MeSH Terms] OR ("samoa"[MeSH Terms] OR "samoa"[All Fields]) OR "Serbia and Montenegro"[All Fields] OR "Sri Lanka"[All Fields] OR "suriname"[MeSH Terms] OR ("suriname"[MeSH Terms] OR "suriname"[All Fields]) OR "swaziland"[MeSH Terms] OR ("swaziland"[MeSH Terms] OR "swaziland"[All Fields]) OR "Syrian Arab Republic"[All Fields] OR "syria"[MeSH Terms] OR ("syria"[MeSH Terms] OR "syria"[All Fields]) OR "thailand"[MeSH Terms] OR ("thailand"[MeSH Terms] OR "thailand"[All Fields]) OR "tonga"[MeSH Terms] OR ("tonga"[MeSH Terms] OR "tonga"[All Fields]) OR "tunisia"[MeSH Terms] OR ("tunisia"[MeSH Terms] OR "tunisia"[All Fields]) OR "turkmenistan"[MeSH Terms] OR ("turkmenistan"[MeSH Terms] OR "turkmenistan"[All Fields]) OR "ukraine"[MeSH Terms] OR ("ukraine"[MeSH Terms] OR "ukraine"[All Fields]) OR "vanuatu"[MeSH Terms] OR ("vanuatu"[MeSH Terms] OR "vanuatu"[All Fields]) OR "West Bank and Gaza"[All Fields] OR "American Samoa"[All Fields] OR "Antigua and Barbuda"[All Fields] OR "argentina"[MeSH Terms] OR ("argentina"[MeSH Terms] OR "argentina"[All Fields]) OR "barbados"[MeSH Terms] OR ("barbados"[MeSH Terms] OR "barbados"[All Fields]) OR "belize"[MeSH Terms] OR ("belize"[MeSH Terms] OR "belize"[All Fields]) OR "botswana"[MeSH Terms] OR ("botswana"[MeSH Terms] OR "botswana"[All Fields]) OR "chile"[MeSH Terms] OR ("chile"[MeSH Terms] OR "chile"[All Fields]) OR "Costa Rica"[All Fields] OR "croatia"[MeSH Terms] OR ("croatia"[MeSH Terms] OR "croatia"[All Fields]) OR "Czech Republic"[All Fields] OR "dominica"[MeSH Terms] OR ("dominica"[MeSH Terms] OR "dominica"[All Fields]) OR "Equatorial Guinea"[All Fields] OR "estonia"[MeSH Terms] OR ("estonia"[MeSH Terms] OR "estonia"[All Fields]) OR "gabon"[MeSH Terms] OR ("gabon"[MeSH Terms] OR "gabon"[All Fields]) OR "grenada"[MeSH Terms] OR ("grenada"[MeSH Terms] OR "grenada"[All Fields]) OR "hungary"[MeSH Terms] OR ("hungary"[MeSH Terms] OR "hungary"[All Fields]) OR "latvia"[MeSH Terms] OR ("latvia"[MeSH Terms] OR "latvia"[All Fields]) OR "lebanon"[MeSH Terms] OR ("lebanon"[MeSH Terms] OR "lebanon"[All Fields]) OR "libya"[MeSH Terms] OR ("libya"[MeSH Terms] OR "libya"[All Fields]) OR "lithuania"[MeSH Terms] OR ("lithuania"[MeSH Terms] OR "lithuania"[All Fields]) OR "malaysia"[MeSH Terms] OR ("malaysia"[MeSH Terms] OR "malaysia"[All Fields]) OR "mauritius"[MeSH Terms] OR ("mauritius"[MeSH Terms] OR "mauritius"[All Fields]) OR "comoros"[All Fields] OR "comoros"[MeSH Terms] OR ("comoros"[MeSH Terms] OR "comoros"[All Fields] OR "mayotte"[All Fields]) OR "mexico"[MeSH Terms] OR ("mexico"[MeSH Terms] OR "mexico"[All Fields]) OR "Northern Mariana Islands"[All Fields] OR "oman"[MeSH Terms] OR ("oman"[MeSH Terms] OR "oman"[All Fields]) OR "palau"[MeSH Terms] OR ("palau"[MeSH Terms] OR "palau"[All Fields]) OR "panama"[MeSH Terms] OR ("panama"[MeSH Terms] OR "panama"[All Fields]) OR "poland"[MeSH Terms] OR ("poland"[MeSH Terms] OR "poland"[All Fields]) OR "Russian Federation"[All Fields] OR "seychelles"[MeSH Terms] OR ("seychelles"[MeSH Terms] OR "seychelles"[All Fields]) OR "Slovak Republic"[All Fields] OR "South Africa"[All Fields] OR "St. Kitts and Nevis"[All Fields] OR "St. Lucia"[All Fields] OR "St. Vincent and the Grenadines"[All Fields] OR "Trinidad and Tobago"[All Fields] OR "turkey"[MeSH Terms] OR ("turkey"[MeSH Terms] OR "turkey"[All Fields]) OR "uruguay"[MeSH Terms] OR ("uruguay"[MeSH Terms] OR "uruguay"[All Fields]) OR "venezuela"[MeSH Terms] OR ("venezuela"[MeSH Terms] OR "venezuela"[All Fields]) OR "developing countries"[All Fields] OR "less developed countries"[All Fields] OR "third-world countries"[All Fields] OR "under-developed countries"[All Fields] OR "poOR countries"[All Fields] OR "less developed countries"[All Fields] OR "under developed countries"[All Fields] OR "less developed nations"[All Fields] OR "third world nations"[All Fields] OR "under developed nations"[All Fields] OR "developing nations"[All Fields] OR "poOR nations"[All Fields] OR "poor economies"[All Fields] OR "developing economies"[All Fields] OR "less developed economies"[All Fields] OR "myanmar"[MeSH Terms] OR ("myanmar"[MeSH Terms] OR "myanmar"[All Fields] OR "burma"[All Fields]) OR "Czechoslovakia"[All Fields] OR "Democratic Republic of Congo"[All Fields] OR "French Guiana"[All Fields] OR "East Timor"[All Fields] OR "laos"[MeSH Terms] OR ("laos"[MeSH Terms] OR "laos"[All Fields]) OR "North Korea"[All Fields] OR "Ivory Coast"[All Fields] OR "Republic of Georgia"[All Fields] OR "Republic of Yemen"[All Fields] OR "Republic of Zaire"[All Fields] OR "slovakia"[MeSH Terms] OR ("slovakia"[MeSH Terms] OR "slovakia"[All Fields]) OR "Soviet Union"[All Fields] OR "suriname"[MeSH Terms] OR ("suriname"[MeSH Terms] OR "suriname"[All Fields] OR "surinam"[All Fields]) OR "ussr"[MeSH Terms] OR ("ussr"[MeSH Terms] OR "ussr"[All Fields]) OR "samoa"[MeSH Terms] OR ("samoa"[MeSH Terms] OR "samoa"[All Fields]) OR "yugoslavia"[MeSH Terms] OR ("yugoslavia"[MeSH Terms] OR "yugoslavia"[All Fields]) OR "democratic republic of the congo"[MeSH Terms] OR ("democratic republic of the congo"[MeSH Terms] OR ("democratic"[All Fields] AND "republic"[All Fields] AND "congo"[All Fields]) OR "democratic republic of the congo"[All Fields] OR "zaire"[All Fields]) OR "asia"[MeSH Terms] OR ("asia"[MeSH Terms] OR "asia"[All Fields]) OR "West Indies"[All Fields] OR "polynesia"[MeSH Terms] OR ("polynesia"[MeSH Terms] OR "polynesia"[All Fields]) OR "micronesia"[MeSH Terms] OR ("micronesia"[MeSH Terms] OR "micronesia"[All Fields]) OR "Middle East"[All Fields] OR "africa"[MeSH Terms] OR ("africa"[MeSH Terms] OR "africa"[All Fields]) OR "Latin America"[All Fields] OR "Central America"[All Fields] OR "South America"[All Fields] OR ("west indies"[MeSH Terms] OR ("west"[All Fields] AND "indies"[All Fields]) OR "west indies"[All Fields]) OR "west indies"[MeSH Terms] OR "caribbean region"[MeSH Terms] OR ("west indies"[MeSH Terms] OR ("west"[All Fields] AND "indies"[All Fields]) OR "west indies"[All Fields] OR "caribbean"[All Fields] OR "caribbean region"[MeSH Terms] OR ("caribbean"[All Fields] AND "region"[All Fields]) OR "caribbean region"[All Fields]) OR "caribbean region"[MeSH Terms] OR Hispanola[All Fields] OR "Southeast Asia"[All Fields] OR "Sub-Saharan Africa"[All Fields] OR "Eastern Europe"[All Fields] OR Balkans[All Fields]) OR "Developing Countries"[MeSH Terms] OR "Developing Country"[All Fields] OR "Developing Countries"[All Fields] OR "Low Resource Setting"[All Fields] OR "Low Resource Settings"[All Fields] OR Bangladesh[mesh] OR bangladesh[all fields])

*1728 hits*

*70 manuscripts pulled*

*14 manuscripts included*

**Research Question 2 (February – August 2010, updated July 2010 - June 2013, updated May 2014)**

***2) What proportion of pharmacies or health facilities have antibiotics available for treating neonatal infections?***

***Antibiotic Search Terms***

["anti-bacterial agents"[MeSH Terms] OR ("anti-bacterial"[All Fields] AND "agents"[All Fields]) OR "anti-bacterial agents"[All Fields] OR "antibiotic"[All Fields] OR "anti-bacterial agents"[Pharmacological Action]

***Search Terms for Access/Availability***

AND [(access OR availability)]

***Search Terms for Developing Country***

AND [Developing Country Terms] as above

*TOTAL – 1364 HITS*

*154 manuscripts pulled*

*7 included*

**Research Questions 3 (June- August 2010, updated July 2010 - June 2013, updated May 2014)**

***3) What proportion of antibiotic purchases for newborns is obtained from over-the-counter mechanisms?***

***Antibiotic Search Terms***

["anti-bacterial agents"[MeSH Terms] OR ("anti-bacterial"[All Fields] AND "agents"[All Fields]) OR "anti-bacterial agents"[All Fields] OR "antibiotic"[All Fields] OR "anti-bacterial agents"[Pharmacological Action] AND

***Over-the-Counter Search Terms***

(((over the counter) OR (self medication) OR (private sector) OR (pharmacy) OR (drug store))

***Neonatal/Infant Terms***

((infant[MeSH] OR (infant[MeSH] OR child[MeSH] OR adolescent[MeSH])))) ]] AND (("infant, newborn"[MeSH Terms] OR ("infant"[All Fields] AND "newborn"[All Fields]) OR "newborn infant"[All Fields] OR "neonatal"[All Fields]) OR (neonat* OR newborn*)))

***Developing Country Terms***

AND [Developing Country Terms] as above

TOTAL – 407 HITS

49 Articles pulled

14 articles Included
