# Supplementary material for: Treatment of Infections in Young Infants in Low- and Middle-Income Countries: A Systematic Review and Meta-analysis of Frontline Health Worker Diagnosis and Antibiotic Access
Source: PLoS Med. 2014 Oct 14;11(10):e1001741. doi: 10.1371/journal.pmed.1001741 (PMC4196753; doi:10.1371/journal.pmed.1001741)
Supplement: Text S3 — Research protocol. (DOC) [file pmed.1001741.s007.doc]

**Review Protocol**

1. **Background**

Neonatal infections result in an estimated 907,000 deaths annually. Case fatality from neonatal infections approaches 40% in low-middle income countries (LMIC), in large part due to poor illness recognition, and inadequate access to medical care, including antibiotics.

1. **Research questions**

1) What proportion of newborns with infection are recognized and prescribed antibiotics by a health provider?

2) What proportion of health facilities or pharmacies have antibiotics available for treating neonatal infections?

3) What proportion of antibiotic purchases for newborns is obtained from over-the-counter mechanisms?

1. **Search Strategy**

This combined automated and manual search will use multiple search engines and databases (see Table 1). Search terms are listed in the Appendix formatted for PubMed.

**Table 1:** Databases and Search engines

| **Database** | **Website** |
| --- | --- |
| PubMed/Medline | <http://www.ncbi.nlm.nih.gov/pubmed> |
| Embase | <http://www.embase.com/> |
| The Cochrane Library | <http://www.cochrane.org/> |
| Global Health Library | <http://www.globalhealth.org/> |
| Eldis | <http://www.eldis.org/> |
| Data Online for Population, Health, and Nutrition | <http://dolphn.aimglobalhealth.org/> |
| Reproductive Health Gateway | <http://www.k4health.org/resources/rhgateway/> |
| Basic Support for Institutionalizing Child Survival | <http://www.basics.org/> |
| Saving Newborn Lives | [http://www.savethechildren.org/site/c.8rKLIXMGIpI4E/b.6234293/k.6211/Saving_Newborn_Lives.htm#](http://www.savethechildren.org/site/c.8rKLIXMGIpI4E/b.6234293/k.6211/Saving_Newborn_Lives.htm) |
| Healthy Newborn Network* | <http://www.healthynewbornnetwork.org/> |
| USAID* | [www.usaid.gov](http://www.usaid.gov/) |
| United Nations* | [www.un.org](http://www.un.org/) |
| UNICEF* | [www.unicef.org](http://www.unicef.org/) |
| World Health Organization* | [www.who.int](http://www.who.int/) |
| Demographic and Health Surveys | <http://www.measuredhs.com/>  [www.statcompiler.com](http://www.statcompiler.com/) |
| Multiple Indicator Cluster Surveys | <http://www.unicef.org/statistics/index_24302.html> |
| Service Provision Assessments | <http://www.measuredhs.com/aboutsurveys/spa/start.cfm> |
| WHO/HAI (Health Action International) | <http://www.haiweb.org/medicineprices/manual/documents.html> |

*Added May 2014.

1. **Selection Criteria**
   1. Inclusion Criteria:
      1. Studies were conducted in LMIC;
      2. Studies describe “antibiotic access” for neonates or children with suspected infection;
      3. Studies with population of newborns or pediatric patients <18 yo
      4. For the above 4 research questions, study specifies either a proportion or a numerator and denominator
   2. Exclusion Criteria:
      1. No language exclusions
      2. Report on adult populations only
      3. Sole focus on “inappropriate” use of antibiotics
      4. Studies from HICs
      5. Studies reporting on antibiotic prescription patterns, or utilization, without an appropriate denominator for the research parameters
      6. Specialized sub-populations
      7. Individual case reports
      8. Duplicate studies
      9. Studies reporting on outbreaks, or susceptibility patterns alone.
2. **Study Quality Assessment**

We will apply basic principles from the Working Group for Grading of Recommendations Assessment, Development and Evaluation (GRADE), with adaptations as described in the Child Health Epidemiology Reference Group reviews (Walker Int J Epidemiol 2010;39(Supplement 1):i21-31) and modified further for the purposes of our research aim. The overall level of evidence for each parameter will be of graded as high, moderate, or low, according to study design, selection bias/population representativeness, definition quality, precision, reporting bias, and generalizability to our population of interest.

Studies’ methods will be examined for appropriate description and completeness. All studies will be extracted by two independent researchers. If the data extractors disagree, they will discuss their position in detail, using evidence from the study in question until they reach a compromise. If they do not reach a compromise, the question at hand will be discussed with the research team during a team meeting to arrive at a compromise that the team as a whole agrees with. Specific quality variables are defined below.

*Selection Bias* will be defined according to the study population selection and representativeness of the general population. A study will be considered to have selection bias if it occurred at a health facility in settings of primarily home birth, at a referral level health facility, or within a specific sub-population.

*Reporting Bias* will be considered in retrospective studies or those relying primarily on parental self-report.

*Consistency* will be defined according to the similarity in estimates and definitions/variables across studies for a given observation or parameter.

*Generalizability* will be considered if the study’s findings may be considered appropriate to apply to the target population of interest (newborns in LMIC).

1. **Data Extraction**

Data will be extracted through the steps outlined in Figure 1 and entered into a data extraction excel file (Table 3).

**Figure 1:** Sample Flowchart for Data Extraction


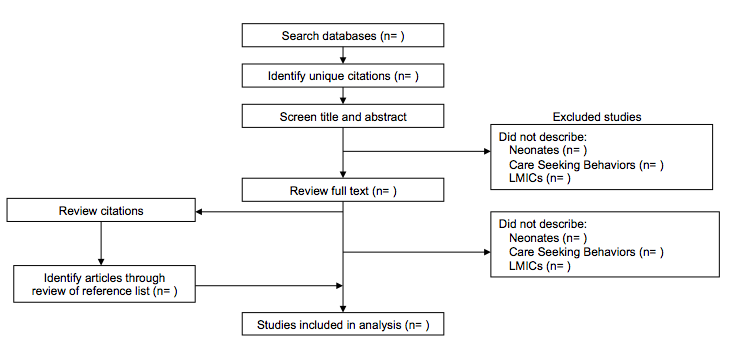


*n denotes the number of studies included in each phase of the data extraction

**Table 2:** Sample Data Extraction Table

| **PI** | **Year** | **Country** | | **WHO Region** | | **Study design** | | **Study setting** | | **Rural/Urban** | | **Age** | | **Population selection** | | **Intervention?** | | | **Sample size** | **Denominator** | | **Prescriber** | | **% prescribed antibiotic** | | **% used antibiotic** | | **% injectable antibiotics** | | **Notes** | |  | |
| --- | --- | --- | --- | --- | --- | --- | --- | --- | --- | --- | --- | --- | --- | --- | --- | --- | --- | --- | --- | --- | --- | --- | --- | --- | --- | --- | --- | --- | --- | --- | --- | --- | --- |
|  | | |  | |  | |  | |  | |  | |  | |  | |  |  | | |  |  |  | |  | |  | |  | |  | |  |

1. **Study Limitations**

The potential limitations we foresee are the paucity of published neonatal data and data relevant for LMIC. We therefore will attempt to target numerous search engines and sources in the grey and unpublished literature. The study may potentially be limited if the studies found in our search are not representative of global regions.

1. **Reporting**

We plan to report these findings to public health experts in child and maternal health through a planned publication in a peer-reviewed journal. This will inform further research and policy to increase antibiotic access for newborns in LMIC.

1. **Project timetable.**

| **Date** | **Objectives** |
| --- | --- |
| January-Feb 2010 | Design, test, and revise search terms |
| Feb- March 2010 | Run searches in database  Upload search results in Refworks**®** |
| March-April 2010 | Screen title and abstract of results |
| April – June 2010 | Obtain full texts of remaining studies and screen for inclusion/exclusion criteria |
| June-September 2010 | Analysis and extract data from studies |
| September – Jan 2011 | Draft primary report of findings |
| January – July 2011 | Internal review and Circulation with Co-authors |
| July 2011 | Submission of Final Report to Bill Melinda Gates Foundation |
| June 2013 | Update all searches |
| July 2013-October 2013 | Analyze new data and Prepare manuscript |
| November | Submit Manuscript for publication |

**10 . Protocol Registration**

The protocol was registered in the PROSPERO International prospective register of systematic reviews, University of York Centre for Reviews and Dissemination (http://www.crd.york.ac.uk/PROSPERO/) (CRD42013004586).
